# Changes in Gut Microbial Composition and DNA Methylation in Obese Patients with NAFLD After Bariatric Surgery

**DOI:** 10.3390/ijms252111510

**Published:** 2024-10-26

**Authors:** Antonella Agodi, Claudia Ojeda-Granados, Andrea Maugeri, Martina Barchitta, Ornella Coco, Salvatore Pezzino, Gaetano Magro, Gaetano La Greca, Francesco Saverio Latteri, Sergio Castorina, Stefano Puleo

**Affiliations:** 1 Department of Medical and Surgical Sciences and Advanced Technologies “GF Ingrassia”, University of Catania, 95123 Catania, Italy; claudiaojedagranados@hotmail.com (C.O.-G.); andrea.maugeri@unict.it (A.M.); martina.barchitta@unict.it (M.B.); ornellacoco19@gmail.com (O.C.); salvatore.pezzino12@virgilio.it (S.P.); gmagro@unict.it (G.M.); glagreca@unict.it (G.L.G.); saverio.latteri@unict.it (F.S.L.); sergio.castorina@unict.it (S.C.); spuleo@unict.it (S.P.); 2 Mediterranean Foundation “GB Morgagni”, 95125 Catania, Italy

**Keywords:** obesity, fatty liver, epigenetic markers, gut microbiota, sleeve gastrectomy, personalized medicine

## Abstract

This study investigates the effects of bariatric surgery on non-alcoholic fatty liver disease (NAFLD) by examining the interplay between gut microbiota, epigenetics, and metabolic health. A cohort of 22 patients undergoing sleeve gastrectomy (SG) was analyzed for changes in gut microbial composition and DNA methylation profiles before and six months after surgery. Correlations between gut microbial abundance and clinical markers at baseline revealed that certain genera were associated with worse metabolic health and liver markers. Following SG, significant improvements were observed in the clinical, anthropometric, and biochemical parameters of the NAFLD patients. Although alpha-diversity indices (i.e., Chao1, Simpson, Shannon) did not show significant changes, beta-diversity analysis revealed a slight shift in microbial composition (PERMANOVA, *p* = 0.036). Differential abundance analysis identified significant changes in specific bacterial taxa, including an increase in beneficial Lactobacillus species such as *Lactobacillus crispatus* and *Lactobacillus iners* and a decrease in harmful taxa like Erysipelotrichia. Additionally, DNA methylation analysis revealed 609 significant differentially methylated CpG sites between the baseline values and six months post-surgery, with notable enrichment in genes related to the autophagy pathway, such as *IRS4* and *ATG4B*. The results highlight the individualized responses to bariatric surgery and underscore the potential for personalized treatment strategies. In conclusion, integrating gut microbiota and epigenetic factors into NAFLD management could enhance treatment outcomes, suggesting that future research should explore microbiome-targeted therapies and long-term follow-ups on liver health post-surgery.

## 1. Introduction

Obesity is the major risk factor for the development of NAFLD (non-alcoholic fatty liver disease), a term used to describe the full spectrum of metabolic liver disorders associated with insulin resistance and dyslipidemia [1]. Initially, NAFLD presents as non-alcoholic hepatic steatosis, which, with persistent or increasing obesity, may progress to non-alcoholic steatohepatitis, characterized by inflammatory lesions that are still reversible if medical and/or surgical interventions (e.g., caloric restriction and metabolic surgery) are employed to treat obesity [2,3,4]. In fact, with a persistent or increasing body mass index (BMI), the risk of progression to advanced liver fibrosis, cirrhosis, and hepatocarcinoma has been shown to increase 4- to 5-fold [5,6,7]. Although obesity and related disorders are the most common conditions associated with NAFLD, this disease can also affect normal-weight individuals [8]. However, because obesity and cardiometabolic dysfunction remain key clinical features of NAFLD, its nomenclature has recently been changed to metabolic-dysfunction-associated steatotic liver disease (MASLD) and steatohepatitis (MASH) [9]. Reaching a consensus on an appropriate definition of this disease is not easy, as the pathophysiology is complex and involves heterogeneous exogenous factors, including dietary and lifestyle factors, and endogenous factors, such as lipogenesis, lipotoxicity, insulin resistance, cell death, and altered gut microbiota [10,11]. In addition, several genetic variants may influence the risk of developing NAFLD (e.g., the rs738409 polymorphism in the *PNPLA3* gene) [12,13,14]. All these factors converge to induce chronic systemic organ inflammation, which particularly fuels the diverse features of NAFLD [15]. Patients with NAFLD also exhibit a different gut microbiota composition compared to healthy individuals [16]. In this regard, there is increasing evidence that the gut–liver axis plays a key role in NAFLD, especially in the progression to more advanced stages of the disease [17,18]. Some studies have also shown an association between *Helicobacter pylori* infection and an increased risk of NAFLD [19]. It has been suggested that *H. pylori* infection may influence the gut microbiota and contribute to the pathogenesis of NAFLD through the action of hormones, bacterial metabolites, alterations in intestinal permeability, and *H. pylori* toxins [19]. Yet, the roles of *H. pylori* and the gut microbiota in the pathogenesis of NAFLD are still not fully known [20]. Targeting the improvement and/or resolution of comorbidities among obese patients, particularly concerning NAFLD, gastric restrictive surgery (e.g., roux-en-Y gastric bypass (RYGB), sleeve gastrectomy (SG)) has been shown to be effective for both weight loss and reducing pro-inflammatory mechanisms [21,22,23]. In particular, weight loss after bariatric surgery has shown to lead to metabolic improvement and the resolution of low-grade systemic inflammation by improving altered metabolic markers such as lipid profile, glucose concentration, insulin levels, and HOMA-IR index and by reducing the concentration of proinflammatory cytokines (e.g., TNF-α, IL-6, IL-8, and IL-1β) [24]. Although not yet fully understood, the response to bariatric surgery procedures may be associated with epigenetic changes, such as changes in DNA methylation signatures that occur following surgery [25]. For example, intraindividual comparison of liver biopsies before and after bariatric surgery demonstrated differential methylation at CpGs sites in loci of the gene encoding protein tyrosine phosphatase epsilon (*PTPRE*), a negative regulator of insulin signaling in skeletal muscle, suggesting that hypermethylation and transcriptional downregulation of *PTPRE* may represent a key mechanism in the recovery of hepatic insulin sensitivity following bariatric surgery [25]. In this study, we analyzed gut microbial composition and DNA methylation profile changes after bariatric surgery in obese patients with NAFLD.

## 2. Results

### 2.1. Characteristics of Study Participants

The clinical characteristics at the baseline (T0) and 6 months post-SG (T1) of the 22 patients with NAFLD included in this study are shown in Table 1. The mean age of the subjects was 44.1 years (range: 19–62 years), and 59.1% were females. Regarding educational level, 4.5% had completed elementary school, 36.4% had completed middle school, and 59.1% had completed high school. In addition, 36.4% were homemakers, and 50.0% were economically inactive, while 36.4% were full-time workers. Among these individuals, 40.9% had type 2 diabetes, and 45.5% had hypertension. All the patients had a hepatic steatosis index (HSI) value > 36, and according to the Fibrosis-4 Index (FIB-4), 86.4% had liver fibrosis stage 0–1, while 13.6% had stage 2–3.

All patients included were sedentary, and 31.8% were smokers, 31.8% were former smokers, and 36.4% were non-smokers. Regarding alcohol intake, 27.2% consumed alcohol rarely or never, 59.1% consumed <11 standard drinks per week, and 13.6% consumed 12–17 standard drinks per week. According to the Short Food Frequency Questionnaire (SFFQ), evaluating the consumption of different foods and nutrients with an important impact on hepatic health, the NAFLD patients showed a mean dietary quality score (DQS) of 113.2 ± 22.9.

### 2.2. Gut Microbiota Diversity and Taxonomic Distribution of Predominant Bacteria in NAFLD Patients

**Figure 1:**
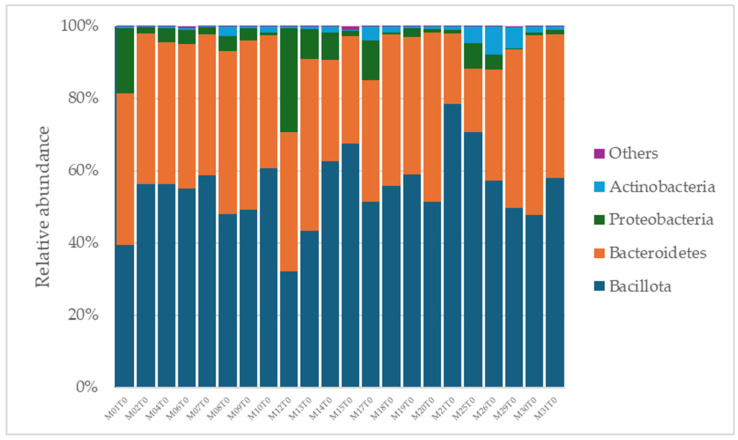
Distribution of phylum-level relative abundance in the gut microbiota of obese patients with NAFLD.

**Figure 2:**
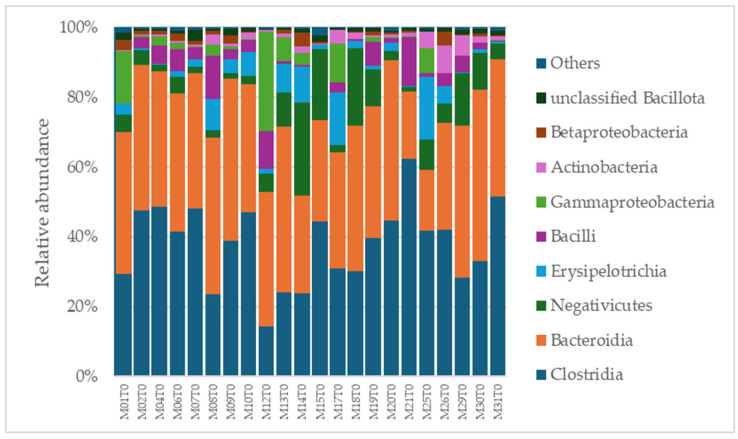
Distribution of class-level relative abundance in the gut microbiota of obese patients with NAFLD.

**Figure 3:**
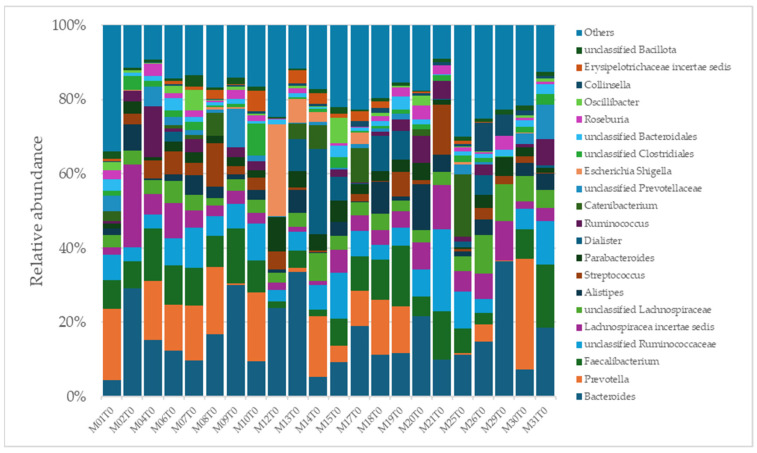
Distribution of genus-level relative abundance in the gut microbiota of obese patients with NAFLD.

A total of 2315 species, 351 genera, 121 families, 64 orders, 37 classes, and 18 phyla were identified in the gut microbiota of the NAFLD patients at T0. The bacterial abundance and distribution of the dominant bacteria at the phylum, class, and genus levels (i.e., relative abundance > 1% of total sequences) are shown in Figure 1, Figure 2 and Figure 3. The most abundant phyla included Bacillota (54.9%), Bacteroidetes (38.0%), Proteobacteria (5.1%), and Actinobacteria (1.8%), which together comprised 99.8% of the total sequences (Figure 1). The predominant classes were Clostridia (38.0%), Bascteroidia (37.7%), Negativicutes (7.7%), Erysipelotrichia (4.3%), Bacilli (3.9%), Gammaproteobacteria (3.8%), Actinobacteria (1.8%), Betaproteobacteria (1.2%), and unclassified Bacillota (1.1%), accounting for 99.4% of the total sequences (Figure 2). As for genera, the most abundant were *Bacteroides* (16.4%), *Prevotella* (8.8%), *Faecalibacterium* (8.5%), unclassified *Ruminococcaceae* (7.2%), *Lachnospiraceae incertae sedis* (5.6%), unclassified *Lachnospiraceae* (4.6%), *Alistipes* (3.4%), *Streptococcus* (3.3%), *Parabacteroides* (3.1%), *Dialister* (3.0%), *Ruminococcus* (2.6%), *Catenibacterium* (2.2%), unclassified *Prevotellaceae* (2.1%), *Escherichia Shigella* (1.8%), unclassified *Clostridiales* (1.7%), unclassified *Bacteroidales* (1.5%), *Roseburia* (1.5%), *Oscillibacter* (1.2%), *Collinsella* (1.1%), *Erysipelotrichaceae incertae sedis* (1.1%), and unclassified *Bacillota* (1.1%), which collectively comprised 81.7% of the total sequences (Figure 3).

### 2.3. Association Between Clinical, Biochemical, and Lifestyle Characteristics and Taxa Relative Abundance of NAFLD Patients at Baseline

**Figure 4:**
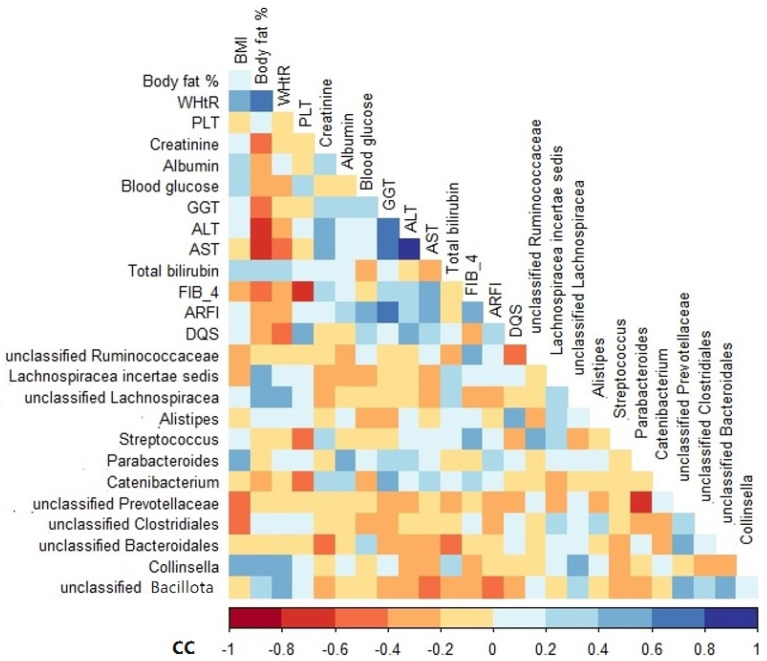
Spearman’s rank correlation coefficient heatmap of the associations between genus relative abundance and clinical variables of NAFLD patients at baseline. Only variables that showed a significant association are shown. BMI, body mass index; WHtR, waist-to-height ratio; PLT, platelet; GGT, gamma-glutamyl transferase; ALT, alanine transaminase; AST, aspartate aminotransferase; HSI, hepatic steatosis index; FIB-4, fibrosis-4 index; ARFI, acoustic radiation force impulse; DQS, dietary quality score; CC, correlation coefficient.

Among the variables analyzed, BMI, body fat, waist-to-height ratio (WHtR), albumin, gamma-glutamyl transferase (GGT), FIB-4, and DQS were positively correlated with the relative abundance of some genera, especially *Parabacteroides*, *Collinsella*, *Lachnospiracea incertae sedis*, unclassified *Lachnospiracea*, unclassified *Bacillota*, *Catenibacterium*, *Streptococcus*, and *Alistipes* (Figure 4). In contrast, the variables that correlated negatively with the relative abundance of unclassified *Clostridiales*, *Catenibacterium*, unclassified *Ruminococcaceae*, unclassified *Bacillota*, and unclassified *Bacteroidales* included BMI, platelet (PLT), blood glucose, aspartate aminotransferase (AST), bilirubin, acoustic radiation force impulse (ARFI), and alcohol intake (Figure 4).

### 2.4. Changes in Clinical Characteristics, Biochemical Parameters, and Gut Microbiota Diversity After Bariatric Surgery

**Figure 5:**
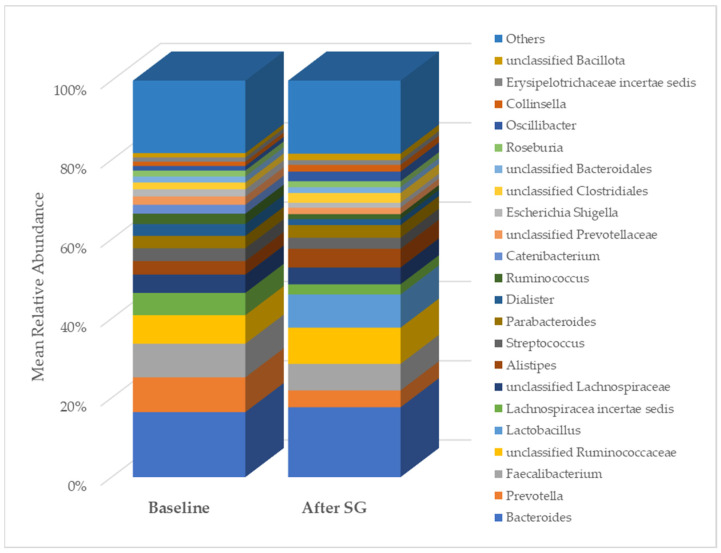
Comparison of genus relative abundance in the gut microbiota between baseline and six months post-bariatric surgery in patients with NAFLD. SG, sleeve gastrectomy.

As expected, after bariatric surgery with SG, most of the clinical, anthropometric, and biochemical parameters of the NAFLD patients changed significantly (Table 1). Regarding gut microbiota diversity, an initial analysis pointed out changes in the relative abundance of some taxa. In particular, at the class level, the relative abundance of Erysipelotrichia, Actinobacteria, and unclassified Bacillota changed after SG. At the genus level, changes were evidenced in the relative abundance of *Prevotella*, *Lachnospiracea incertae sedis*, and unclassified *Bacillota* (Figure 5).

**Figure 6:**
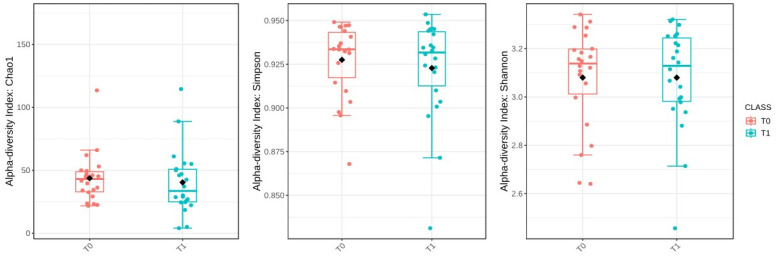
Comparison of alpha-diversity indices (Chao1, Simpson, and Shannon) between baseline (T0) and after sleeve gastrectomy (T1). The black diamond represents the mean value.

Then, the alpha-diversity of the gut microbiota was evaluated to assess microbial diversity within individual samples at the baseline (T0) and six months post-surgery (T1). However, statistical comparisons revealed no significant changes in alpha-diversity between these two time points. The Chao1 index, which estimates species richness, had a *p*-value of 0.541; the Simpson index, which measures species dominance, had a *p*-value of 0.650; and the Shannon index, which reflects both richness and evenness, had a *p*-value of 0.991 (Figure 6).

**Figure 7:**
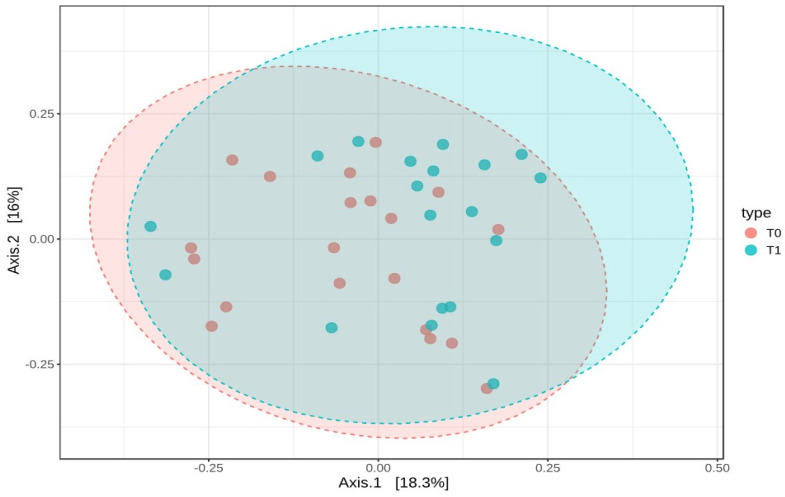
Beta-diversity analysis using Bray–Curtis dissimilarity and PCoA plot of microbial composition at T0 and T1. This figure represents the beta-diversity analysis based on Bray–Curtis dissimilarity, along with a Principal Coordinates Analysis (PCoA) plot visualizing the microbial composition at baseline (T0) and six months post-surgery (T1).

These results suggest that the microbial diversity within the samples remained stable over time. To examine how the composition of the gut microbiota varied between T0 and T1, beta-diversity was assessed using the Bray–Curtis dissimilarity index. A PERMANOVA test was conducted to determine if the overall microbial community composition differed significantly between the two time points. The analysis showed a statistically significant difference, with an F-value of 1.8737, an R-squared of 0.0482, and a *p*-value of 0.036. However, despite this statistical significance, the PCoA plot based on Bray–Curtis distances did not reveal distinct clusters, indicating that while there were compositional changes, the shifts were not substantial enough to clearly separate the two groups (Figure 7).

To further investigate which specific bacterial taxa contributed to the observed differences between T0 and T1, a differential abundance analysis was performed. This analysis revealed significant changes across multiple taxonomic levels, from class to species (Table 2). At the class level, Bacilli showed a significant increase in abundance post-surgery, with a log2 fold change (log2FC) of 2.8717, while Erysipelotrichia displayed a significant decrease, with a log2FC of −1.2383. At the order level, the Lactobacillales order was significantly enriched at T1, with a log2FC of 2.8717. At the family level, the Lactobacillaceae family showed a marked increase in abundance post-surgery, with a log2FC of 3.09. At the genus level, the *Lactobacillus* genus was significantly enriched at T1, with a log2FC of 3.0832. Finally, at the species level, two *Lactobacillus* species exhibited significant increases post-surgery, namely, *Lactobacillus crispatus*, with a log2FC of 1.6327, and *Lactobacillus iners*, with a log2FC of 1.365.

### 2.5. Changes in the DNA Methylation Profile After Bariatric Surgery

**Figure 8:**
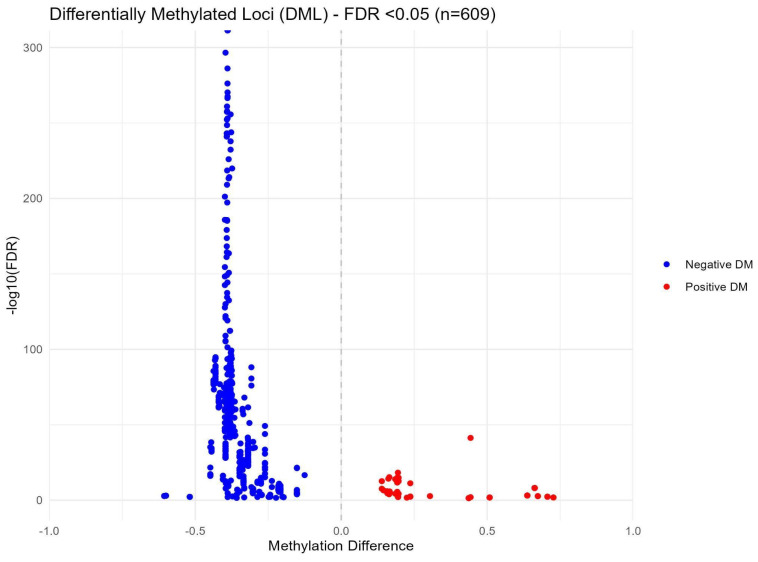
Volcano plot of methylation differences between T0 and T1 in paired samples.

After sequencing quality control checks were conducted, seven samples and their corresponding pairs were excluded from further analysis due to insufficient read counts (M01T0, M06T0, M09T0, M15T1, M19T0, M30T0, and M31T0). The remaining samples underwent a rigorous quality control assessment using MultiQC, confirming adequate coverage and quality for subsequent methylation analyses. In the group-based comparison, where all samples from time points T0 and T1 were pooled, no statistically significant differences were detected. However, the pair-wise differential methylation analysis revealed 609 significant differences (with a False Discovery Rate (FDR) < 0.05) across 550 distinct CpG sites between the two time points. Details are reported in the Supplementary Materials (Table S1). Among these differences, 98 CpG sites exhibited increased methylation, while 511 CpG sites demonstrated decreased methylation. Figure 8 illustrates these methylation changes through a volcano plot, highlighting significantly differentially methylated loci in red and blue to indicate positive and negative differential methylation, respectively.

**Figure 9:**
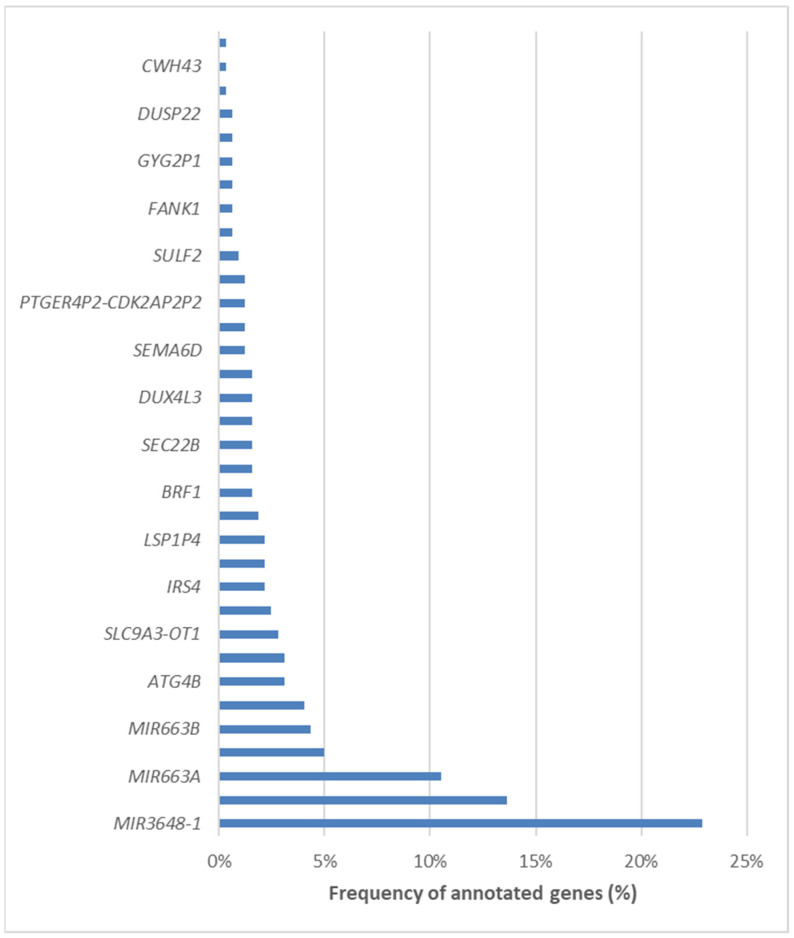
List of the 34 differentially methylated genes in NAFLD patients undergoing bariatric surgery.

We subsequently annotated the differentially methylated CpG loci and mapped them to specific genomic regions. Remarkably, 423 loci were located within promoter regions, 73 were mapped to introns, and 40 were identified in distal intergenic regions. A comprehensive list of the 34 involved genes can be found in Figure 9. Among the differentially methylated genes, three stood out as the most frequently represented, i.e., *MIR3648-1*, *PMF1*, and *MIR663A*.

Functional annotation analysis using the Kyoto Encyclopedia of Genes and Genomes (KEGG) database identified that the only significantly enriched pathway associated with the differentially methylated genes was “Autophagy-animal” (hsa04140). This pathway is crucial for maintaining cellular homeostasis through the degradation and recycling of cellular components. Within this pathway, two key genes were identified: *Insulin Receptor Substrate 4* (*IRS4*) and *Autophagy-related 4B Cysteine Peptidase* (*ATG4B*). *IRS4* plays a significant role in insulin signaling and has implications in metabolic regulation, while *ATG4B* is essential for the formation of autophagosomes, facilitating the autophagy process.

## 3. Discussion

The findings of this study provide important insights into how bariatric surgery impacts obese patients with NAFLD, revealing significant changes in clinical markers, gut microbiota composition, and DNA methylation patterns. These changes underscore the multifaceted benefits of SG on metabolic health, with potential implications for the treatment of NAFLD.

The significant reductions in BMI, body fat percentage, and WHtR observed post-surgery confirm the efficacy of SG in reducing obesity and its associated metabolic risks. The improvements observed in key biochemical markers, such as GGT, ALT, AST, and triglycerides, further indicate substantial improvements in liver function and overall metabolic health following SG. These results are consistent with the findings of previous studies, which have demonstrated that bariatric surgery can mitigate liver inflammation and reverse steatosis in patients with NAFLD [26,27]. However, the slight progression in liver fibrosis, as indicated by the FIB-4 index in a small percentage of patients, is an unexpected finding. Although this increase was modest, it raises important questions about the long-term effects of rapid weight loss on liver tissue remodeling. Some studies suggest that initial fibrosis progression may be part of a transient remodeling process post-surgery, but these patients require continued monitoring to ensure their fibrosis does not advance further [28,29].

The gut microbiota plays a critical role in regulating metabolic health and liver function, particularly through the gut–liver axis [30]. At the baseline, the gut microbiota of the NAFLD patients was dominated by the Bacillota and Bacteroidetes phyla, consistent with the dysbiosis often observed in obesity and metabolic disorders [31]. Following SG, there were decreases in potentially harmful taxa, such as Erysipelotrichia and unclassified *Bacillota*, alongside an increase in *Prevotella*, a genus linked to improved metabolic outcomes [32,33]. These findings suggest that SG not only facilitates weight loss but also contributes to the restoration of a healthier gut microbial balance, which may play a key role in enhancing overall metabolic health. These initial observations highlight the critical involvement of the gut–liver axis in NAFLD and suggest that SG may improve liver function not only through weight loss but also by promoting a more favorable gut microbiome. For example, positive associations were found between harmful genera such as *Collinsella*, *Parabacteroides*, and *Streptococcus* and clinical markers of metabolic dysfunction, including elevated BMI, body fat, and GGT levels. These associations suggest that these taxa may contribute to both metabolic disturbances and liver inflammation [34,35,36]. Conversely, negative correlations were observed between beneficial taxa such as *Ruminococcaceae* and improved liver markers (e.g., AST, bilirubin), underscoring the potential protective role of certain bacteria in liver health [37].

Interestingly, despite the major physiological changes induced by SG, our analysis of alpha-diversity indices (Chao1, Simpson, and Shannon) showed no significant differences between the baseline (T0) and six months post-surgery (T1). This indicates that while SG influences the metabolic environment, it does not significantly alter the overall richness or evenness of the gut microbiota within individual samples. This finding is consistent with previous studies, which have similarly reported little to no change in alpha-diversity following bariatric surgery [38,39]. This suggests that the impact of SG on the microbiota may be more focused on specific bacterial taxa rather than overall community diversity. Similarly, although beta-diversity analysis revealed a statistically significant change in microbial composition between T0 and T1, the PCoA plot did not show clearly separated clusters. This also suggests that while there were compositional changes, they were not substantial enough to create distinct microbial profiles across patients. These subtle shifts may reflect changes in key taxa that affect metabolic health without dramatically altering the overall community structure.

One of the most striking findings in our study is the significant increase in *Lactobacillus* and related taxa following surgery. Across multiple taxonomic levels, including class, order, family, genus, and species, members of the Lactobacillaceae family—especially *Lactobacillus crispatus* and *Lactobacillus iners*—showed significant enrichment after SG. This aligns with findings from previous studies that also reported an increase in *Lactobacillus* after bariatric procedures [40,41]. The post-surgical enrichment of *Lactobacillus* could be explained by changes in the gastrointestinal environment following SG, including alterations in pH, bile acid composition, and nutrient availability [40]. Lactobacilli are known to thrive in acidic environments and play a crucial role in gut health by producing lactic acid and supporting gut barrier integrity. Moreover, the increased presence of *Lactobacillus* may contribute to improving post-surgical metabolic regulation, as certain *Lactobacillus* species have been linked to enhanced insulin sensitivity and anti-inflammatory effects [42]. The enrichment of *Lactobacillus crispatus* and *Lactobacillus iners* specifically could have important implications for the management of NAFLD in patients undergoing bariatric surgery. Previous research suggests that some strains of *Lactobacillus* may influence liver function by reducing inflammation and modulating lipid metabolism [42], thus potentially playing a role in the positive metabolic outcomes often observed after SG.

These observations highlight the critical involvement of the gut–liver axis in NAFLD and suggest that bariatric surgery may improve liver health not only through weight loss but also by promoting a healthier gut microbiome [37]. The identification of specific microbial taxa linked to liver disease markers raises the possibility of using microbiome-targeted interventions—such as probiotics, prebiotics, or fecal microbiota transplantation—as potential adjunct therapies for NAFLD [43]. Future clinical trials could explore whether restoring a balanced gut microbiota, in conjunction with bariatric surgery, enhances liver recovery and metabolic outcomes more effectively. Additionally, these studies should delve into the functional implications of microbiota changes after surgery, focusing on microbial metabolites like short-chain fatty acids, bile acids, and other bioactive compounds [44]. Understanding the mechanisms by which gut bacteria regulate liver function and influence NAFLD progression could shed light on novel therapeutic strategies targeting the gut–liver axis, ultimately optimizing the treatment and management of NAFLD.

Epigenetic modifications, particularly DNA methylation, play a critical role in how environmental factors, such as diet and lifestyle changes, influence gene expression and overall metabolic health. In this study, nearly 600 differentially methylated CpG sites were identified when comparing samples before and six months after bariatric surgery, with most of them showing reduced methylation. These alterations indicate that bariatric surgery induces significant epigenetic reprogramming, which likely contributed to the observed metabolic improvements. One of the most notable findings was the enrichment of differentially methylated genes within the “Autophagy-animal” pathway, a key process responsible for protein degradation, organelle turnover, and the breakdown of cytoplasmic components. Autophagy is tightly regulated by environmental stressors such as nutrient deprivation, growth factor withdrawal, and endoplasmic reticulum stress, playing a vital role in maintaining cellular homeostasis and quality control [45]. An important function of autophagy in NAFLD is the regulation of the process of excessive lipid accumulation. For example, mice fed high-fat diets and in which an autophagy-related gene (*Atg7*) was knocked out showed a marked increase in hepatic triglycerides and cholesterol content, indicating that defects in autophagy may promote hepatic steatosis [46]. In addition, insulin downregulates autophagy in response to nutrient supply, but autophagy also modulates insulin sensitivity. As expected, hyperinsulinemic mice fed high-fat diets have shown reduced levels of autophagy [47]. Thus, independent factors can promote both impaired autophagy and hepatic steatosis, but then decreased autophagy exacerbates steatosis, further impairing autophagy. This complex cycle leads to a persistent worsening of both cellular autophagic function and lipid accumulation [48]. The post-surgical methylation changes in autophagy-related genes, including *IRS4* and *ATG4B*, suggest a restoration of this critical process. *IRS4* is involved in insulin signaling, and *ATG4B* plays a role in autophagosome formation [49,50]—two mechanisms that could explain how SG improves insulin sensitivity and reduces hepatic fat accumulation. In addition to the autophagy-related genes, other frequently represented differentially methylated genes, such as *MIR3648-1*, *PMF1*, and *MIR663A*, are involved in processes like cell proliferation, differentiation, and inflammation [51,52,53]. Their altered methylation patterns highlight their potential role as key regulators of the metabolic changes observed after surgery and warrant further investigation into their complication in the epigenetic regulation of obesity-related liver disease. To build on these findings, transcriptomic data could be integrated to provide a deeper understanding of how DNA methylation impacts gene expression and metabolic regulation. This approach could help identify the regulatory pathways that mediate the beneficial effects of bariatric surgery on liver function, offering new insights into therapeutic targets for NAFLD and related metabolic conditions.

Several limitations must be acknowledged when interpreting these results. First, this study’s small sample size of 22 patients limits its statistical power and generalizability. With a cohort of this size, it is not possible to account for multiple confounding factors, such as diet, BMI, medications, and physical activity, which are known to influence both gut microbiota composition and epigenetic modifications. Consequently, some of the observed changes may be partly attributable to these unmeasured variables. Another limitation is that all the participants underwent SG and shared similar metabolic conditions, restricting the applicability of the findings to other types of bariatric surgery, such as RYGB, or to individuals with varying severities of NAFLD. Future research should include larger and more diverse patient populations, as well as comparisons between different surgical procedures, to determine whether the observed changes in microbiota and clinical markers are specific to SG or generalizable to other bariatric interventions. Furthermore, the six-month follow-up period may not have been long enough to allow a full assessment of the long-term effects of SG, particularly with regard to liver fibrosis and the sustainability of gut microbiota changes. Given that fibrosis is a slow-progressing condition, a longer follow-up is needed to determine whether the improvements observed in this study are maintained or continue to evolve beyond six months.

Longer-term studies with robust controls for dietary and lifestyle factors are essential to determine the persistence of these improvements and assess the potential for fibrosis reversal over extended periods. Lastly, this study did not include a control group of NAFLD patients who did not undergo surgery, which limits the ability to attribute the observed changes solely to the effects of bariatric surgery. Future studies should incorporate control groups to compare the natural progression of NAFLD with the post-surgical outcomes, ensuring a clearer understanding of how surgery specifically alters disease trajectory. Furthermore, although significant changes in microbial composition and DNA methylation were identified, this study did not delve into the functional implications of these alterations, such as the production of microbial metabolites or the specific genes regulated by differentially methylated loci. Integrating advanced techniques such as metagenomics, transcriptomics, and metabolomics in future research could provide a more comprehensive view of the functional consequences of these epigenetic and microbial shifts, leading to a better understanding of the mechanisms driving metabolic improvement after surgery.

## 4. Materials and Methods

### 4.1. Study Design and Population

In this epidemiological study, approved by the Catania Ethics Committee (Catania 2, Prot. No. 110, 28 September 2022, and Prot. No. 600, 28 March 2023), we recruited patients with NAFLD at two medical centers in Catania city: Policlinico Morgagni and Azienda Ospedaliera Cannizzaro. The present quasi-experimental, one-group pretest–posttest design study included patients who underwent bariatric surgery at Policlinico Morgagni from November 2022 to September 2023. In particular, subjects ≥ 18 years old with obesity (class I, II, or III) and NAFLD preliminarily assessed by changes in liver function and liver ultrasound and who were eligible for bariatric surgery according to the guidelines of the Italian Society of Obesity Surgery (SICOB) were included [54]. In accordance with routine clinical practice, patients underwent esophagogastroduodenoscopy with biopsy for *H. pylori* testing. *H. pylori*-positive patients were not included in this study and were referred for appropriate eradication therapy. Clinical characteristics, biochemical parameters, gut microbiota diversity, and DNA methylation profiles were assessed to compare changes between baseline (T0) and after six months of bariatric surgery (T1). The association between the clinical, biochemical, and lifestyle characteristics and gut microbiota compositions of the subjects at T0 was also analyzed. All participants signed an informed consent form.

### 4.2. Clinical and Biochemical Assessment

Biochemical parameters assessed for all participants included PLT count, urea, creatinine, albumin, blood glucose, total cholesterol, triglycerides, high-density lipoprotein cholesterol (HDL-c), low-density lipoprotein cholesterol (LDL-c), very-low-density lipoprotein cholesterol (VLDL-c), GGT, alanine transaminase (ALT), AST, AST/ALT ratio, and total bilirubin. HSI was evaluated according to the formula 8 × (ALT/AST) + BMI + 2 (if female) + 2 (if type 2 diabetes) and considering a cut-off value < 30 to exclude and >36 to predict hepatic steatosis [55]. In addition, the FIB-4 was used to assess liver fibrosis. FIB-4 was calculated as age (years) × AST (IU/L)/[PLT (10^9^/L) × √ALT (IU/L)] [56]. Accordingly, the approximate fibrosis stage was defined according to the Ishak fibrosis staging cut-off points, considering values < 1.45 as stage 0–1, in the 1.45–3.25 range as stage 2–3, and >3.25 as stage 4–6 liver fibrosis [56]. Acoustic Radiation Force Impulse (ARFI) elastography was also performed using a Siemens Acuson ultrasound unit (Siemens Healthineers AG, Munich, Germany) to assess liver stiffness and determine liver fibrosis stage. Body weight and fat percentage were measured via bioelectrical impedance analysis. BMI was calculated as body weight (Kg) divided by the square of height (m) to define the subjects’ nutritional status according to the World Health Organization classification [57].

### 4.3. Lifestyle and Diet Quality Assessment

An ad hoc questionnaire was prepared to collect sociodemographic (i.e., age, gender, educational level, profession, and employment status), clinical (i.e., disease history, use of medications/supplements/stimulants), and lifestyle (i.e., smoking habit, physical activity level) data of study participants. In addition, overall diet quality was assessed at baseline using a relatively validated SFFQ for NAFLD patients [58,59]. With the authors’ permission, the SFFQ was translated into Italian. In particular, this instrument assesses the frequency of consumption per week, over the last month, of food from the fruit, vegetable, sugar, and fat groups, including alcohol consumption, which is particularly implicated in NAFLD pathogenesis. Each question is scored on a scale of 0 to 5, with a score of 5 indicating optimal intake, and the sum of all points leads to a DQS [58,59].

### 4.4. Bariatric Surgery Intervention and Follow-Up of Patients

Patients recruited at Policlinico Morgagni underwent bariatric surgery with SG. The clinical characteristics, biochemical parameters, gut microbiota diversity, and DNA methylation profiles of patients were assessed to compare changes at the 6-month follow-up after bariatric surgery. For this purpose, stool and blood samples were collected from fasting patients at the time of admission, prior to undergoing SG, and again during the follow-up visit, six months post-surgery.

### 4.5. Gut Microbiota Analysis

To estimate the diversity of the patients’ gut microbial communities, the V1–V2–V3 hypervariable regions of the bacterial 16S rRNA gene were amplified and sequenced. Stool samples were collected at baseline (T0) and 6 months after (T1) SG for all NAFLD patients and stored at −80 °C until analysis. After initial lysis buffer processing, DNA extraction was performed by using the MagPurix^®^ Bacterial DNA Extraction Kit and the MagPurix^®^ Automated Extraction System (Resnova, Rome, Italy) according to the manufacturer’s instructions. DNA purity was measured using a NanoDrop 1000 spectrophotometer (ThermoFisher Scientific, Waltham, MA, USA), and DNA concentration was determined using a Qubit™ fluorometer with the Qubit™ dsDNA High-Sensitivity Assay (HS) Kit (ThermoFisher Scientific, Waltham, MA, USA). For library preparation, Microbiota Solution A (Arrow Diagnostics S.R.L., Genova, Italy) was used to target and analyze the V1–V2–V3 hypervariable regions of the bacterial 16S rRNA gene. This kit utilizes degenerated primers, which allow for the amplification of multiple hypervariable regions, increasing the ability to capture a broader range of bacterial diversity. All procedures were performed in accordance with the manufacturer’s instructions. Library quality controls were performed using both the Qubit™ dsDNA HS kit and the High Sensitivity D1000 ScreenTape assay for TapeStation (Agilent Technologies, Santa Clara, CA, USA) to assess concentration, profile, and size of each library obtained. Next-generation sequencing was performed in paired-end mode (2 × 151 bp) on a Mid Output v2 platform for Illumina NextSeq^®^ 550 (Illumina, San Diego, CA, USA). Variants were called with a variant allele frequency cut-off value of 30%. Target regions showed a mean read coverage of 60× (10% quantile) with a minimum depth of >25× for 99% of bases. Sequencing data (FASTQ files) were analyzed using the software MicrobAT Suite v1.2.1 (SmartSeq S.R.L., Novara, Italy) that identified operational taxonomic units. The raw counts for each taxon were extracted and analyzed across multiple taxonomic levels (e.g., genus, family, species).

Alpha-diversity, which reflects the diversity within individual samples, was evaluated using three widely applied indices: the Chao1 Index, which estimates species richness within each sample; the Shannon Index, which accounts for both richness (number of species) and evenness (distribution of individuals across species); and the Simpson Index, which emphasizes species dominance within a sample. These indices were calculated using the QIIME2 pipeline. Statistical comparisons of alpha-diversity between groups (T0 versus T1) were made using the Wilcoxon signed-rank test, appropriate for paired sample comparisons. Beta-diversity, which assesses differences in microbial community composition between samples, was evaluated using the Bray–Curtis dissimilarity index, which quantifies the compositional dissimilarity based on species abundance. To test for significant differences in microbial community composition between groups, we applied PERMANOVA (Permutational Multivariate Analysis of Variance). To further explore and visualize these differences, Principal Coordinates Analysis (PCoA) was performed on the Bray–Curtis distance matrix.

For the Differential Abundance Analysis, the EdgeR package was employed to identify taxa that were significantly different between groups. Raw counts were normalized using the Trimmed Mean of M-values (TMM) method to account for variations in sequencing depth. Taxa with consistently low counts across samples were filtered out to minimize noise. A negative binomial generalized linear model (GLM) was then used to model the count data, and significance testing was performed using the likelihood ratio test (LRT). Multiple testing was corrected using the Benjamini–Hochberg (BH) method to control the false discovery rate (FDR), with taxa showing adjusted *p*-values < 0.05 being considered significantly differentially abundant. Results were reported as log2 fold changes (log2FC), alongside raw *p*-values and FDR-adjusted *p*-values.

### 4.6. DNA Methylation Analysis

To evaluate changes in the DNA methylation profiles between T0 and T1, peripheral blood samples were collected in EDTA tubes and stored at −80 °C until analysis. Genomic DNA was extracted using the magnetic-bead-based MagPurix^®^ Blood DNA Extraction Kit and the MagPurix^®^ Automated Extraction System (Resnova, Rome, Italy) according to the manufacturer’s instructions. DNA concentrations were measured with a Qubit™ fluorometer using the Qubit™ dsDNA HS Kit (ThermoFisher Scientifc, Waltham, MA, USA). Reduced Representation Bisulfite Sequencing (RRBS) was performed on a total of 46 samples (i.e., samples at T0 and T1 and controls) using the Zymo-Seq RRBS Kit (Zymo Research Corporation, Irvine, CA, USA) according to the manufacturer’s protocol. Libraries were sequenced on four Illumina NextSeq^®^ 550 runs (Illumina, San Diego, CA, USA), generating paired-end reads. Raw sequencing data (FASTQ files) were analyzed using a bioinformatics pipeline tailored to bisulfite sequencing data. Adapter trimming and quality control checks were first performed with Trim Galore v0.6.6 (Babraham Bioinformatics, Cambridge, UK) according to the Zymo-Seq RRBS Kit protocol guidelines. Quality control of trimmed sequences was performed using FastQC v0.11.9 (Babraham Bioinformatics, Cambridge, UK) to assess sequence quality metrics. The curated reads were aligned to the human genome assembly GRCh37 (hg19) using the Bismark aligner v0.22.3 (Babraham Bioinformatics, Cambridge, UK). A comprehensive quality control analysis was performed, using MultiQC v1.18 (Seqera Labs, Barcelona, Spain) to subsequently perform differentially methylated loci (DML) analysis. A Group-Based Comparison (GBC) analysis was performed by pooling all T0 and T1 samples. CpG sites with a minimum read coverage of 10× were included, and differences in methylation levels were analyzed using Fisher’s exact test, using the methylKit package v1.20.0 [60] in Rstudio v4.3.3. Paired-Sample Analysis (PSA) was also performed, comparing methylation levels between T0 and T1 paired samples. CpG sites within a ±50 base pair window around each locus were analyzed. Significant differences in methylation levels were analyzed using Welch’s *t*-test (*p*-value < 0.05), followed by post hoc correction with the Benjamini–Hochberg method. Only sites with a False Discovery Rate (FDR) < 0.05 were retained as reliable DML. These analyses were performed using the DSS package [61] in Rstudio v4.3.3. CpG site annotation was performed using the ChIPseeker package v1.28.0 [62] in combination with the TxDb.Hsapiens.UCSC.hg19.knownGene database for gene identification. The ggplot2 package in R was used to generate volcano plots to represent genome-wide DML patterns and highlight loci with statistically significant methylation differences between T0 and T1.

### 4.7. Statistical Analysis

Quantitative variables were expressed as medians and interquartile ranges, while categorical variables were given as frequencies and percentages. Spearman’s rank correlation was used to analyze the association between clinical, biochemical, and lifestyle variables and taxa relative abundance at baseline. The Wilcoxon signed-rank test was used to analyze changes in clinical and biochemical quantitative variables after SG. The McNemar’s test or marginal homogeneity test was implemented to analyze changes in categorical variables with two or more categories. These analyses were performed with SPSS software (v 22.0, SPSS Inc., Chicago, IL, USA), considering a *p*-value < 0.05 as statistically significant.

## 5. Conclusions

This study highlights the intricate nature of NAFLD, emphasizing the complex interplay between gut microbiota, epigenetics, and metabolic health. Bariatric surgery not only drives significant weight loss and improves liver function but also may contribute to profound shifts in both microbial communities and epigenetic landscapes. Importantly, these changes appear to be highly individualized, reflecting unique responses at the microbial and epigenetic levels. Given this complexity, future research should focus on developing personalized treatment strategies that integrate genetic, epigenetic, and microbial profiles to optimize outcomes for NAFLD patients. This multifactorial approach—addressing this disease through both surgical and biological pathways—provides new insights into the mechanisms underlying NAFLD progression and resolution. By considering an individual’s baseline microbiota, genetic susceptibility, and epigenetic markers, tailored interventions could significantly improve both short- and long-term outcomes for NAFLD management. In light of these findings, NAFLD patients undergoing bariatric surgery may benefit from personalized treatment plans that not only target weight loss but also address microbial and epigenetic factors. Future research should explore the potential of combining bariatric surgery with microbiome-based therapies and epigenetic interventions to maximize the therapeutic effects. Long-term studies are also needed to assess the sustainability of these changes and evaluate whether liver fibrosis can be effectively reversed in post-surgical NAFLD patients.

## Figures and Tables

**Table 1:** Clinical characteristics and biochemical parameters of NAFLD patients included in the study.

Clinical Parameter	NAFLD Patients Undergoing SG (*n* = 22)		*p*
	Baseline	After SG	
BMI, kg/m^2^	40.4 (5.6)	30.5 (4.8)	<0.001
Overweight	0 (0.0)	8 (36.4)	<0.001
Obesity Class I	0 (0.0)	10 (45.5)	
Obesity Class II	10 (45.5)	3 (13.6)	
Obesity Class III	12 (54.5)	1 (4.5)	
Body fat, %	65.0 (22.0)	46.8 (18.0)	<0.001
WHtR	0.78 (0.06)	0.69 (0.10)	<0.001
PLT, 10^9^/L	262.0 (78.0)	222.5 (77.0)	<0.001
Urea, mg/dL	31.4 (7.3)	30.9 (10.3)	0.626
Creatinine, mg/dL	0.78 (0.22)	0.71 (0.20)	0.062
Albumin, g/dL	4.4 (0.5)	4.3 (0.4)	0.650
Blood glucose, mg/dL	90.0 (19)	86.5 (9.0)	0.030
Total cholesterol, mg/dL	189.5 (43)	182.4 (49.0)	0.455
Triglycerides, mg/dL	118.0 (83.0)	84.0 (41.0)	<0.001
HDL-c, mg/dL	45.0 (11.0)	48.5 (17.0)	0.010
LDL-c, mg/dL	119.5 (32.0)	113.05 (33.0)	0.263
VLDL-c, mg/dL	23.6 (16.5)	16.8 (8.1)	<0.001
GGT, IU/L	28.0 (23.0)	13.0 (8.0)	<0.001
ALT, IU/L	25.5 (29.3)	11.4 (10.1)	<0.001
AST, IU/L	20.9 (11.9)	16.0 (7.5)	<0.001
AST/ALT ratio	0.74 (0.34)	1.3 (0.75)	<0.001
Total bilirubin, mg/dL	0.37 (0.25)	0.67 (0.33)	<0.001
HIS	54.2 (9.8)	39.1 (6.55)	<0.001
FIB-4	0.65 (0.44)	0.79 (0.63)	<0.001
FIB-4 fibrosis stage			0.317
0–1	19 (86.4)	18 (81.8)	
2–3	3 (13.6)	4 (18.2)	
ARFI fibrosis stage			0.317
F0	19 (86.4)	18 (81.8)	
F1	3 (13.6)	3 (13.6)	
F2	0 (0.0)	1 (4.5)	

Variables are expressed as medians (interquartile ranges) or frequencies (percentages). SG, sleeve gastrectomy; BMI, body mass index; WHtR, waist-to-height ratio; PLT, platelet; HDL-c, high-density lipoprotein cholesterol; LDL-c, low-density lipoprotein cholesterol; VLDL-c, very-low-density lipoprotein cholesterol; GGT, gamma-glutamyl transferase; ALT, alanine transaminase; AST, aspartate aminotransferase; HSI, hepatic steatosis index; FIB-4, fibrosis-4 index; ARFI, acoustic radiation force impulse.

**Table 2:** Summary of Differential Abundance Analysis (DAA) across taxonomic levels before and after sleeve gastrectomy.

Taxonomy Level	Name	log2FC	*p*-Values	FDR
Class	Bacilli	2.8717	3.08 × 10^−6^	8.63 × 10^−5^
Class	Erysipelotrichia	−1.2383	0.002898	0.04057
Order	Lactobacillales	2.8717	3.0069 × 10^−6^	0.000153
Family	Lactobacillaceae	3.09	6.6511 × 10^−7^	5.7865 × 10^−5^
Genus	Lactobacillus	3.0832	4.9059 × 10^−7^	0.000106
Species	*Lactobacillus crispatus*	1.6327	1.1462 × 10^−7^	9.2617 × 10^−5^
Species	*Lactobacillus iners*	1.365	2.4339 × 10^−5^	0.01311

This table summarizes the differential abundance of microbial taxa at various taxonomic levels (class, order, family, genus, species) between baseline (T0) and six months post-surgery (T1), highlighting significant log2 fold changes and corresponding *p*-values and false discovery rate (FDR)-adjusted *p*-values.

## Data Availability

Research data can be shared upon request.

## Supplementary Materials

The following supporting information can be downloaded at https://www.mdpi.com/article/10.3390/ijms252111510/s1.

## References

[1] Kani H.T., Ozer Demirtas C., Keklikkiran C., Ergenc I., Mehdiyev S., Akdeniz E., Yilmaz Y. (2021). Evaluation of the Impact of Metabolic Syndrome on Fibrosis in Metabolic Dysfunction-Associated Fatty Liver Disease. Turk. J. Gastroenterol..

[2] Friedman S.L., Neuschwander-Tetri B.A., Rinella M., Sanyal A.J. (2018). Mechanisms of NAFLD Development and Therapeutic Strategies. Nat. Med..

[3] Esquivel C.M., Garcia M., Armando L., Ortiz G., Lascano F.M., Foscarini J.M. (2018). Laparoscopic Sleeve Gastrectomy Resolves NAFLD: Another Formal Indication for Bariatric Surgery?. Obes. Surg..

[4] Fakhry T.K., Mhaskar R., Schwitalla T., Muradova E., Gonzalvo J.P., Murr M.M. (2019). Bariatric Surgery Improves Nonalcoholic Fatty Liver Disease: A Contemporary Systematic Review and Meta-Analysis. Surg. Obes. Relat. Dis..

[5] Sepulveda-Villegas M., Roman S., Rivera-Iñiguez I., Ojeda-Granados C., Gonzalez-Aldaco K., Torres-Reyes L.A., Jose-Abrego A., Panduro A. (2019). High Prevalence of Nonalcoholic Steatohepatitis and Abnormal Liver Stiffness in a Young and Obese Mexican Population. PLoS ONE.

[6] Petrucciani N., Gugenheim J. (2019). Molecular Pathways between Obesity, Non-Alcoholic Steatohepatitis (NASH) and Hepatocellular Carcinoma (HCC). Hepatobiliary Surg. Nutr..

[7] Marengo A., Rosso C., Bugianesi E. (2016). Liver Cancer: Connections with Obesity, Fatty Liver, and Cirrhosis. Annu. Rev. Med..

[8] Seo M.-W., Eum Y., Jung H.C. (2023). Increased Risk of Cardiometabolic Disease in Normal-Weight Individuals with Non-Alcoholic Fatty Liver Disease. Obes. Res. Clin. Pract..

[9] Rinella M.E., Lazarus J.V., Ratziu V., Francque S.M., Sanyal A.J., Kanwal F., Romero D., Abdelmalek M.F., Anstee Q.M., Arab J.P. (2024). A Multisociety Delphi Consensus Statement on New Fatty Liver Disease Nomenclature. Ann. Hepatol..

[10] Tilg H., Adolph T.E., Dudek M., Knolle P. (2021). Non-Alcoholic Fatty Liver Disease: The Interplay between Metabolism, Microbes and Immunity. Nat. Metab..

[11] Eslam M., Newsome P.N., Sarin S.K., Anstee Q.M., Targher G., Romero-Gomez M., Zelber-Sagi S., Wai-Sun Wong V., Dufour J.-F., Schattenberg J.M. (2020). A New Definition for Metabolic Dysfunction-Associated Fatty Liver Disease: An International Expert Consensus Statement. J. Hepatol..

[12] Mazo D.F., Malta F.M., Stefano J.T., Salles A.P.M., Gomes-Gouvea M.S., Nastri A.C.S., Almeida J.R., Pinho J.R.R., Carrilho F.J., Oliveira C.P. (2019). Validation of PNPLA3 Polymorphisms as Risk Factor for NAFLD and Liver Fibrosis in an Admixed Population. Ann. Hepatol..

[13] Salari N., Darvishi N., Mansouri K., Ghasemi H., Hosseinian-Far M., Darvishi F., Mohammadi M. (2021). Association between PNPLA3 Rs738409 Polymorphism and Nonalcoholic Fatty Liver Disease: A Systematic Review and Meta-Analysis. BMC Endocr. Disord..

[14] Pirola C.J., Salatino A., Quintanilla M.F., Castaño G.O., Garaycoechea M., Sookoian S. (2022). The Influence of Host Genetics on Liver Microbiome Composition in Patients with NAFLD. eBioMedicine.

[15] Furman D., Campisi J., Verdin E., Carrera-Bastos P., Targ S., Franceschi C., Ferrucci L., Gilroy D.W., Fasano A., Miller G.W. (2019). Chronic Inflammation in the Etiology of Disease across the Life Span. Nat. Med..

[16] Yang C., Xu J., Xu X., Xu W., Tong B., Wang S., Ji R., Tan Y., Zhu Y. (2023). Characteristics of Gut Microbiota in Patients with Metabolic Associated Fatty Liver Disease. Sci. Rep..

[17] Aron-Wisnewsky J., Warmbrunn M.V., Nieuwdorp M., Clément K. (2020). Nonalcoholic Fatty Liver Disease: Modulating Gut Microbiota to Improve Severity?. Gastroenterology.

[18] Sharpton S.R., Schnabl B., Knight R., Loomba R. (2021). Current Concepts, Opportunities, and Challenges of Gut Microbiome-Based Personalized Medicine in Nonalcoholic Fatty Liver Disease. Cell Metab..

[19] Castaño-Rodríguez N., Mitchell H.M., Kaakoush N.O. (2017). NAFLD, Helicobacter Species and the Intestinal Microbiome. Best Pract. Res. Clin. Gastroenterol..

[20] Cheng D., He C., Ai H., Huang Y., Lu N. (2017). The Possible Role of Helicobacter Pylori Infection in Non-Alcoholic Fatty Liver Disease. Front. Microbiol..

[21] von Schönfels W., Beckmann J.H., Ahrens M., Hendricks A., Röcken C., Szymczak S., Hampe J., Schafmayer C. (2018). Histologic Improvement of NAFLD in Patients with Obesity after Bariatric Surgery Based on Standardized NAS (NAFLD Activity Score). Surg. Obes. Relat. Dis..

[22] Lassailly G., Caiazzo R., Buob D., Pigeyre M., Verkindt H., Labreuche J., Raverdy V., Leteurtre E., Dharancy S., Louvet A. (2015). Bariatric Surgery Reduces Features of Nonalcoholic Steatohepatitis in Morbidly Obese Patients. Gastroenterology.

[23] Kwak M., Mehaffey J.H., Hawkins R.B., Hsu A., Schirmer B., Hallowell P.T. (2020). Bariatric Surgery Is Associated with Reduction in Non-Alcoholic Steatohepatitis and Hepatocellular Carcinoma: A Propensity Matched Analysis. Am. J. Surg..

[24] Villarreal-Calderon J.R., Cuellar-Tamez R., Castillo E.C., Luna-Ceron E., García-Rivas G., Elizondo-Montemayor L. (2021). Metabolic Shift Precedes the Resolution of Inflammation in a Cohort of Patients Undergoing Bariatric and Metabolic Surgery. Sci. Rep..

[25] Ahrens M., Ammerpohl O., von Schönfels W., Kolarova J., Bens S., Itzel T., Teufel A., Herrmann A., Brosch M., Hinrichsen H. (2013). DNA Methylation Analysis in Nonalcoholic Fatty Liver Disease Suggests Distinct Disease-Specific and Remodeling Signatures after Bariatric Surgery. Cell Metab..

[26] Kheirvari M., Dadkhah Nikroo N., Jaafarinejad H., Farsimadan M., Eshghjoo S., Hosseini S., Anbara T. (2020). The Advantages and Disadvantages of Sleeve Gastrectomy; Clinical Laboratory to Bedside Review. Heliyon.

[27] Głuszyńska P., Łukaszewicz A., Diemieszczyk I., Chilmończyk J., Reszeć J., Citko A., Szczerbiński Ł., Krętowski A., Razak Hady H. (2023). The Effect of Laparoscopic Sleeve Gastrectomy on the Course of Non-Alcoholic Fatty Liver Disease in Morbidly Obese Patients during One Year of Follow Up. J. Clin. Med..

[28] Eilenberg M., Langer F.B., Beer A., Trauner M., Prager G., Staufer K. (2018). Significant Liver-Related Morbidity After Bariatric Surgery and Its Reversal-a Case Series. Obes. Surg..

[29] Ahmed L., Gebran S., Persaud A., Saeed K., Khan K., Saeed S., Alothman S., Passos-Fox B., DePaz H., Suman P. (2023). The Use of Noninvasive Scores in Predicting NAFLD Progression After Bariatric Surgery. Obes. Surg..

[30] Wu J., Wang K., Wang X., Pang Y., Jiang C. (2021). The Role of the Gut Microbiome and Its Metabolites in Metabolic Diseases. Protein Cell.

[31] Hassan N.E., El-Masry S.A., El Shebini S.M., Ahmed N.H., Mohamed T.F., Mostafa M.I., Afify M.A.S., Kamal A.N., Badie M.M., Hashish A. (2024). Gut Dysbiosis Is Linked to Metabolic Syndrome in Obese Egyptian Women: Potential Treatment by Probiotics and High Fiber Diets Regimen. Sci. Rep..

[32] Kaakoush N.O. (2015). Insights into the Role of Erysipelotrichaceae in the Human Host. Front. Cell. Infect. Microbiol..

[33] Tett A., Pasolli E., Masetti G., Ercolini D., Segata N. (2021). Prevotella Diversity, Niches and Interactions with the Human Host. Nat. Rev. Microbiol..

[34] Ley R.E., Turnbaugh P.J., Klein S., Gordon J.I. (2006). Human Gut Microbes Associated with Obesity. Nature.

[35] Turnbaugh P.J., Hamady M., Yatsunenko T., Cantarel B.L., Duncan A., Ley R.E., Sogin M.L., Jones W.J., Roe B.A., Affourtit J.P. (2009). A Core Gut Microbiome in Obese and Lean Twins. Nature.

[36] Le Chatelier E., Nielsen T., Qin J., Prifti E., Hildebrand F., Falony G., Almeida M., Arumugam M., Batto J.-M., Kennedy S. (2013). Richness of Human Gut Microbiome Correlates with Metabolic Markers. Nature.

[37] Hsu C.L., Schnabl B. (2023). The Gut-Liver Axis and Gut Microbiota in Health and Liver Disease. Nat. Rev. Microbiol..

[38] Park Y.S., Ahn K., Yun K., Jeong J., Baek K.-W., Lee J., Kim H.-H., Han K., Ahn Y.J. (2023). Alterations in Gastric and Gut Microbiota Following Sleeve Gastrectomy in High-Fat Diet-Induced Obese Rats. Sci. Rep..

[39] Lin X.-H., Huang K.-H., Chuang W.-H., Luo J.-C., Lin C.-C., Ting P.-H., Young S.-H., Fang W.-L., Hou M.-C., Lee F.-Y. (2018). The Long Term Effect of Metabolic Profile and Microbiota Status in Early Gastric Cancer Patients after Subtotal Gastrectomy. PLoS ONE.

[40] Ulker İ., Yildiran H. (2019). The Effects of Bariatric Surgery on Gut Microbiota in Patients with Obesity: A Review of the Literature. Biosci. Microbiota Food Health.

[41] Mohammadzadeh N., Razavi S., Ebrahimipour G. (2024). Impact of Bariatric Surgery on Gut Microbiota Composition in Obese Patients Compared to Healthy Controls. AMB Expr..

[42] Dempsey E., Corr S.C. (2022). *Lactobacillus* Spp. for Gastrointestinal Health: Current and Future Perspectives. Front. Immunol..

[43] Hitch T.C.A., Hall L.J., Walsh S.K., Leventhal G.E., Slack E., de Wouters T., Walter J., Clavel T. (2022). Microbiome-Based Interventions to Modulate Gut Ecology and the Immune System. Mucosal Immunol..

[44] Mann E.R., Lam Y.K., Uhlig H.H. (2024). Short-Chain Fatty Acids: Linking Diet, the Microbiome and Immunity. Nat. Rev. Immunol..

[45] Allen E.A., Baehrecke E.H. (2020). Autophagy in Animal Development. Cell Death Differ..

[46] Yang L., Li P., Fu S., Calay E.S., Hotamisligil G.S. (2010). Defective Hepatic Autophagy in Obesity Promotes ER Stress and Causes Insulin Resistance. Cell Metab..

[47] Liu H.-Y., Han J., Cao S.Y., Hong T., Zhuo D., Shi J., Liu Z., Cao W. (2009). Hepatic Autophagy Is Suppressed in the Presence of Insulin Resistance and Hyperinsulinemia: Inhibition of FoxO1-Dependent Expression of Key Autophagy Genes by Insulin. J. Biol. Chem..

[48] Czaja M.J., Ding W.-X., Donohue T.M., Friedman S.L., Kim J.-S., Komatsu M., Lemasters J.J., Lemoine A., Lin J.D., Ou J.J. (2013). Functions of Autophagy in Normal and Diseased Liver. Autophagy.

[49] Choi E., Bai X.-C. (2023). The Activation Mechanism of the Insulin Receptor: A Structural Perspective. Annu. Rev. Biochem..

[50] Xiong H., Sun L., Lian J., He F. (2023). Involvement of Acetylation of ATG4B in Controlling Autophagy Induction. Autophagy.

[51] Rashid F., Awan H.M., Shah A., Chen L., Shan G. (2017). Induction of miR-3648 Upon ER Stress and Its Regulatory Role in Cell Proliferation. Int. J. Mol. Sci..

[52] Wang S., Liu J., Li C., Yang X. (2017). Research of the Effect of miR-663 on the Proliferation of Prostate Cancer Cells, and the Correlations of miR-663 with Pathological Grade and Clinical Stage. J. BU ON.

[53] Li Y., Pan Y., Zhao X., Wu S., Li F., Wang Y., Liu B., Zhang Y., Gao X., Wang Y. (2024). Peroxisome Proliferator-Activated Receptors: A Key Link between Lipid Metabolism and Cancer Progression. Clin. Nutr..

[54] De Luca M., Zappa M.A., Zese M., Bardi U., Carbonelli M.G., Carrano F.M., Casella G., Chianelli M., Chiappetta S., Iossa A. (2022). Development of the Italian Clinical Practice Guidelines on Bariatric and Metabolic Surgery: Design and Methodological Aspects. Nutrients.

[55] Lee J.-H., Kim D., Kim H.J., Lee C.-H., Yang J.I., Kim W., Kim Y.J., Yoon J.-H., Cho S.-H., Sung M.-W. (2010). Hepatic Steatosis Index: A Simple Screening Tool Reflecting Nonalcoholic Fatty Liver Disease. Dig. Liver Dis..

[56] Sterling R.K., Lissen E., Clumeck N., Sola R., Correa M.C., Montaner J., S Sulkowski M., Torriani F.J., Dieterich D.T., Thomas D.L. (2006). Development of a Simple Noninvasive Index to Predict Significant Fibrosis in Patients with HIV/HCV Coinfection. Hepatology.

[57] Weir C.B., Jan A. (2024). BMI Classification Percentile and Cut Off Points. StatPearls.

[58] Bredin C., Naimimohasses S., Norris S., Wright C., Hancock N., Hart K., Moore J.B. (2020). Development and Relative Validation of a Short Food Frequency Questionnaire for Assessing Dietary Intakes of Non-Alcoholic Fatty Liver Disease Patients. Eur. J. Nutr..

[59] Naimimohasses S., O’Gorman P., Wright C., Ni Fhloinn D., Holden D., Conlon N., Monaghan A., Kennedy M., Gormley J., Beddy P. (2022). Differential Effects of Dietary versus Exercise Intervention on Intrahepatic MAIT Cells and Histological Features of NAFLD. Nutrients.

[60] Akalin A., Kormaksson M., Li S., Garrett-Bakelman F.E., Figueroa M.E., Melnick A., Mason C.E. (2012). methylKit: A Comprehensive R Package for the Analysis of Genome-Wide DNA Methylation Profiles. Genome Biol..

[61] Park Y., Wu H. (2016). Differential Methylation Analysis for BS-Seq Data under General Experimental Design. Bioinformatics.

[62] Yu G., Wang L.-G., He Q.-Y. (2015). ChIPseeker: An R/Bioconductor Package for ChIP Peak Annotation, Comparison and Visualization. Bioinformatics.

