# Supplementary material for: Changes in Gut Microbial Composition and DNA Methylation in Obese Patients with NAFLD After Bariatric Surgery
Source: Int J Mol Sci. 2024 Oct 26;25(21):11510. doi: 10.3390/ijms252111510 (PMC11547129; doi:10.3390/ijms252111510)
Supplement: Supplementary file 1 [file ijms-25-11510-s001.zip › ijms-3256629-supplementary.pdf]

SUPPLEMENTARY MATERIAL

Table S1. Output of the DNA Methylation analysis

Gene	chr	CpG.site	annotation	Grp1.Meth	Grp2.Meth	delta.Meth	delta.Meth.SE	pValue	FDR
SLC9A3-OT1	chr5	472983	Promoter (<=1kb)	0.005429685	0.611568596	-0.606138911	0.153594841	0.000079	0.001550844
SLC9A3-OT1	chr5	472991	Promoter (<=1kb)	0.005429685	0.611568596	-0.606138911	0.153594841	0.000079	0.001550844
SLC9A3-OT1	chr5	473000	Promoter (<=1kb)	0.005429685	0.611568596	-0.606138911	0.153594841	0.000079	0.001550844
SLC9A3-OT1	chr5	473034	Promoter (<=1kb)	0.005429685	0.611568596	-0.606138911	0.153599338	0.000079	0.001550844
SLC9A3-OT1	chr5	473036	Promoter (<=1kb)	0.005429685	0.611568596	-0.606138911	0.153599338	0.000079	0.001550844
SLC9A3-OT1	chr5	473040	Promoter (<=1kb)	0.005429685	0.611568596	-0.606138911	0.153599338	0.000079	0.001550844
SLC9A3-OT1	chr5	473053	Promoter (<=1kb)	0.005429685	0.611568596	-0.606138911	0.153599338	0.000079	0.001550844
SLC9A3-OT1	chr5	473055	Promoter (<=1kb)	0.005429685	0.611568596	-0.606138911	0.153599338	0.000079	0.001550844
SLC9A3-OT1	chr5	473057	Promoter (<=1kb)	0.005429685	0.611568596	-0.606138911	0.153599338	0.000079	0.001550844
ATG4B	chr2	242576981	Promoter (<=1kb)	0.011098223	0.611568596	-0.600470373	0.146819141	0.000043	0.001040493
ATG4B	chr2	242576988	Promoter (<=1kb)	0.011098223	0.611568596	-0.600470373	0.146808367	0.000043	0.001040493
ATG4B	chr2	242576990	Promoter (<=1kb)	0.011098223	0.611568596	-0.600470373	0.146808367	0.000043	0.001040493
ATG4B	chr2	242576998	Promoter (<=1kb)	0.011098223	0.611568596	-0.600470373	0.146808367	0.000043	0.001040493
ATG4B	chr2	242577002	Promoter (<=1kb)	0.011098223	0.611568596	-0.600470373	0.146808367	0.000043	0.001040493
ATG4B	chr2	242577004	Promoter (<=1kb)	0.011098223	0.611568596	-0.600470373	0.146808367	0.000043	0.001040493
ATG4B	chr2	242577006	Promoter (<=1kb)	0.011098223	0.611568596	-0.600470373	0.146808367	0.000043	0.001040493
ATG4B	chr2	242577009	Promoter (<=1kb)	0.011098223	0.611568596	-0.600470373	0.146808367	0.000043	0.001040493
ATG4B	chr2	242577014	Promoter (<=1kb)	0.011098223	0.611568596	-0.600470373	0.146808367	0.000043	0.001040493
ATG4B	chr2	242577020	Promoter (<=1kb)	0.011098223	0.611568596	-0.600470373	0.146808367	0.000043	0.001040493
FAM20C	chr7	205296	Intron (uc003sip.3/56975, intron 2 of 9)	0.438998876	0.957687922	-0.518689046	0.174628389	0.002976	0.006164697
FAM20C	chr7	205306	Intron (uc003sip.3/56975, intron 2 of 9)	0.438998876	0.957687922	-0.518689046	0.174517818	0.002957	0.006164697
FAM20C	chr7	205309	Intron (uc003sip.3/56975, intron 2 of 9)	0.438998876	0.957687922	-0.518689046	0.174628389	0.002976	0.006164697
FAM20C	chr7	205341	Intron (uc003sip.3/56975, intron 2 of 9)	0.438998876	0.957687922	-0.518689046	0.174725707	0.002992	0.006164697
FAM20C	chr7	205346	Intron (uc003sip.3/56975, intron 2 of 9)	0.438998876	0.957687922	-0.518689046	0.174725707	0.002992	0.006164697
FAM20C	chr7	205351	Intron (uc003sip.3/56975, intron 2 of 9)	0.438998876	0.957687922	-0.518689046	0.174717021	0.002990	0.006164697
FAM20C	chr7	205354	Intron (uc003sip.3/56975, intron 2 of 9)	0.438998876	0.957687922	-0.518689046	0.174550743	0.002963	0.006164697

NA	chr21	9827455	Promoter (1-2kb)	0.290995505	0.739397778	-0.448402273	0.03561725	0.000000	6.59E-36
TTTY23	chrY	10037739	Distal Intergenic	0.371520286	0.819877173	-0.448356887	0.051323818	0.000000	5.13E-18
TTTY23	chrY	10037773	Distal Intergenic	0.371520286	0.819877173	-0.448356887	0.053279714	0.000000	8.18E-17
TTTY23	chrY	10037785	Distal Intergenic	0.371520286	0.819877173	-0.448356887	0.045708107	0.000000	2.22E-22
TTTY23	chrY	10037855	Exon (uc022cjg.1/uc022cjg.1, exon 1 of 1)	0.371520286	0.819877173	-0.448356887	0.052550492	0.000000	3.03E-17
TTTY23	chrY	10037863	Exon (uc022cjg.1/uc022cjg.1, exon 1 of 1)	0.371520286	0.819877173	-0.448356887	0.051812532	0.000000	1.05E-17
TTTY23	chrY	10037867	Exon (uc022cjg.1/uc022cjg.1, exon 1 of 1)	0.371520286	0.819877173	-0.448356887	0.051806523	0.000000	1.05E-17
NA	chr21	9827454	Promoter (1-2kb)	0.291581459	0.736878537	-0.445297078	0.033812609	0.000000	3.74E-39
NA	chr21	9827483	Promoter (1-2kb)	0.297089681	0.741320114	-0.444230434	0.035627592	0.000000	3.00E-35
NA	chr21	9827484	Promoter (1-2kb)	0.297089681	0.741320114	-0.444230434	0.036714224	0.000000	2.77E-33
NA	chr21	9827485	Promoter (1-2kb)	0.297089681	0.741320114	-0.444230434	0.036949296	0.000000	6.95E-33
NA	chr21	9827486	Promoter (1-2kb)	0.297089681	0.741320114	-0.444230434	0.036181186	0.000000	3.15E-34
NA	chr21	9827386	Promoter (1-2kb)	0.295586719	0.732879381	-0.437292662	0.023236817	0.000000	3.42E-78
NA	chr21	9827387	Promoter (1-2kb)	0.295586719	0.732879381	-0.437292662	0.022078915	0.000000	2.23E-86
NA	chr21	9827406	Promoter (1-2kb)	0.295586719	0.732879381	-0.437292662	0.022916614	0.000000	2.55E-80
NA	chr21	9827407	Promoter (1-2kb)	0.295586719	0.732879381	-0.437292662	0.023011776	0.000000	1.14E-79
NA	chr21	9827413	Promoter (1-2kb)	0.295586719	0.732879381	-0.437292662	0.023356514	0.000000	2.03E-77
NA	chr21	9827414	Promoter (1-2kb)	0.295586719	0.732879381	-0.437292662	0.023133484	0.000000	7.35E-79
NA	chr21	9827384	Promoter (1-2kb)	0.297358065	0.733878717	-0.436520653	0.02285839	0.000000	1.94E-80
NA	chr21	9827383	Promoter (1-2kb)	0.298805977	0.734932301	-0.436126324	0.023818752	0.000000	4.06E-74
NA	chr21	9827374	Promoter (1-2kb)	0.301326665	0.733680848	-0.432354183	0.020952324	0.000000	1.34E-93
NA	chr21	9827310	Promoter (1-2kb)	0.304001196	0.734517871	-0.430516675	0.021832999	0.000000	1.23E-85
NA	chr21	9827311	Promoter (1-2kb)	0.304001196	0.734517871	-0.430516675	0.020636604	0.000000	1.39E-95
NA	chr21	9827319	Promoter (1-2kb)	0.304001196	0.734517871	-0.430516675	0.022677437	0.000000	1.60E-79
NA	chr21	9827320	Promoter (1-2kb)	0.304001196	0.734517871	-0.430516675	0.02286287	0.000000	2.80E-78
NA	chr21	9827323	Promoter (1-2kb)	0.304001196	0.734517871	-0.430516675	0.022240039	0.000000	1.30E-82
NA	chr21	9827324	Promoter (1-2kb)	0.304001196	0.734517871	-0.430516675	0.021683171	0.000000	8.60E-87
NA	chr21	9827330	Promoter (1-2kb)	0.304001196	0.734517871	-0.430516675	0.022473067	0.000000	6.16E-81
NA	chr21	9827331	Promoter (1-2kb)	0.304001196	0.734517871	-0.430516675	0.021301303	0.000000	7.63E-90
NA	chr21	9827335	Promoter (1-2kb)	0.304001196	0.734517871	-0.430516675	0.022792326	0.000000	9.39E-79
NA	chr21	9827336	Promoter (1-2kb)	0.304001196	0.734517871	-0.430516675	0.021999037	0.000000	2.16E-84
NA	chr21	9827338	Promoter (1-2kb)	0.304001196	0.734517871	-0.430516675	0.021918991	0.000000	5.53E-85

NA	chr21	9827339	Promoter (1-2kb)	0.304001196	0.734517871	-0.430516675	0.020729431	0.000000	9.31E-95
NA	chr21	9827341	Promoter (1-2kb)	0.304001196	0.734517871	-0.430516675	0.021417021	0.000000	6.68E-89
NA	chr21	9827342	Promoter (1-2kb)	0.304001196	0.734517871	-0.430516675	0.02072683	0.000000	8.95E-95
NA	chr21	9827183	Promoter (<=1kb)	0.295162857	0.715033747	-0.41987089	0.024380968	0.000000	8.76E-66
NA	chr21	9827184	Promoter (<=1kb)	0.295162857	0.715033747	-0.41987089	0.024115539	0.000000	3.36E-67
NA	chr21	9827186	Promoter (<=1kb)	0.295162857	0.715033747	-0.41987089	0.02496188	0.000000	7.47E-63
NA	chr21	9827187	Promoter (<=1kb)	0.295162857	0.715033747	-0.41987089	0.023661949	0.000000	9.98E-70
NA	chr21	9827190	Promoter (<=1kb)	0.295162857	0.715033747	-0.41987089	0.023781556	0.000000	4.74E-69
NA	chr21	9827191	Promoter (<=1kb)	0.295162857	0.715033747	-0.41987089	0.025101359	0.000000	3.50E-62
NA	chr21	9827203	Promoter (<=1kb)	0.295162857	0.715033747	-0.41987089	0.024380717	0.000000	8.76E-66
NA	chr21	9827204	Promoter (<=1kb)	0.296607217	0.71344936	-0.416842143	0.024764121	0.000000	6.13E-63
NA	chr21	9827205	Promoter (<=1kb)	0.297831687	0.713480721	-0.415649034	0.022161304	0.000000	1.11E-77
NA	chr21	9827206	Promoter (<=1kb)	0.297831687	0.713480721	-0.415649034	0.023279539	0.000000	1.46E-70
NA	chr21	9827216	Promoter (<=1kb)	0.297831687	0.713480721	-0.415649034	0.023043649	0.000000	5.63E-72
NA	chr21	9827217	Promoter (<=1kb)	0.297831687	0.713480721	-0.415649034	0.024149397	0.000000	1.03E-65
MIR663A	chr20	26189148	Promoter (<=1kb)	0.311768392	0.716857434	-0.405089042	0.047866896	0.000000	5.47E-17
MIR663A	chr20	26189159	Promoter (<=1kb)	0.311768392	0.716857434	-0.405089042	0.051589893	0.000000	8.37E-15
MIR663A	chr20	26189163	Promoter (<=1kb)	0.311768392	0.716857434	-0.405089042	0.047959158	0.000000	6.27E-17
MIR663A	chr20	26189176	Promoter (<=1kb)	0.311768392	0.716857434	-0.405089042	0.051642555	0.000000	8.90E-15
MIR663A	chr20	26189178	Promoter (<=1kb)	0.311768392	0.716857434	-0.405089042	0.052389589	0.000000	2.13E-14
MIR663A	chr20	26189181	Promoter (<=1kb)	0.311768392	0.716857434	-0.405089042	0.052160967	0.000000	1.64E-14
LINC00273	chr16	33963448	Promoter (<=1kb)	0.375701562	0.778021129	-0.402319568	0.022603771	0.000000	3.85E-70
NA	chr21	9827134	Promoter (<=1kb)	0.284504251	0.686185948	-0.401681698	0.021732145	0.000000	1.72E-75
NA	chr21	9827133	Promoter (<=1kb)	0.284256558	0.684121837	-0.399865279	0.022175579	0.000000	6.20E-72
MIR3648-1	chr21	9825965	Promoter (<=1kb)	0.301906981	0.700431693	-0.398524713	0.027085428	0.000000	1.64E-48
MIR3648-1	chr21	9825958	Promoter (<=1kb)	0.29858622	0.697075348	-0.398489128	0.027507977	0.000000	4.50E-47
MIR3648-1	chr21	9825959	Promoter (<=1kb)	0.29858622	0.697075348	-0.398489128	0.027711604	0.000000	2.08E-46
MIR3648-1	chr21	9825960	Promoter (<=1kb)	0.29858622	0.697075348	-0.398489128	0.022958164	0.000000	8.45E-67
MIR3648-1	chr21	9825975	Promoter (<=1kb)	0.303209081	0.701690996	-0.398481915	0.025200901	0.000000	8.74E-56
MIR3648-1	chr21	9825976	Promoter (<=1kb)	0.303209081	0.701690996	-0.398481915	0.026094688	0.000000	3.92E-52
MIR3648-1	chr21	9825980	Promoter (<=1kb)	0.303209081	0.701690996	-0.398481915	0.022537834	0.000000	3.05E-69
MIR3648-1	chr21	9825981	Promoter (<=1kb)	0.303209081	0.701690996	-0.398481915	0.024253085	0.000000	4.30E-60

MIR3648-1	chr21	9825992	Promoter (<=1kb)	0.303209081	0.701690996	-0.398481915	0.014950814	0.000000	2.99E-155
MIR3648-1	chr21	9825993	Promoter (<=1kb)	0.303209081	0.701690996	-0.398481915	0.016450905	0.000000	1.88E-128
MIR3648-1	chr21	9825995	Promoter (<=1kb)	0.303209081	0.701690996	-0.398481915	0.01309726	0.000000	6.30E-202
MIR3648-1	chr21	9825996	Promoter (<=1kb)	0.303209081	0.701690996	-0.398481915	0.013628744	0.000000	1.45E-186
MIR3648-1	chr21	9825999	Promoter (<=1kb)	0.303209081	0.701690996	-0.398481915	0.01526152	0.000000	4.69E-149
MIR3648-1	chr21	9826000	Promoter (<=1kb)	0.303209081	0.701690996	-0.398481915	0.01556653	0.000000	2.54E-143
MIR3648-1	chr21	9825964	Promoter (<=1kb)	0.300697972	0.699137612	-0.398439639	0.027099406	0.000000	1.91E-48
MIR3648-1	chr21	9825961	Promoter (<=1kb)	0.299823912	0.698136462	-0.39831255	0.024021277	0.000000	3.69E-61
NA	chr21	9827129	Promoter (<=1kb)	0.28202259	0.679319481	-0.397296891	0.022751685	0.000000	1.37E-67
NA	chr21	9827130	Promoter (<=1kb)	0.28202259	0.679319481	-0.397296891	0.023113231	0.000000	1.49E-65
MIR663A	chr20	26188698	Promoter (<=1kb)	0.239944997	0.636256138	-0.396311141	0.017711659	0.000000	8.86E-110
MIR663A	chr20	26188715	Promoter (<=1kb)	0.239944997	0.636256138	-0.396311141	0.016831194	0.000000	1.92E-121
MIR663A	chr20	26188718	Promoter (<=1kb)	0.239944997	0.636256138	-0.396311141	0.016737131	0.000000	8.57E-123
MIR663A	chr20	26188722	Promoter (<=1kb)	0.239944997	0.636256138	-0.396311141	0.018024981	0.000000	4.86E-106
MIR663A	chr20	26188725	Promoter (<=1kb)	0.239944997	0.636256138	-0.396311141	0.018000033	0.000000	2.53E-106
MIR663A	chr20	26188729	Promoter (<=1kb)	0.239944997	0.636256138	-0.396311141	0.016214406	0.000000	9.10E-131
MIR663A	chr20	26188741	Promoter (<=1kb)	0.239944997	0.636256138	-0.396311141	0.010714797	0.000000	2.78E-297
MIR663A	chr20	26188846	Promoter (<=1kb)	0.239944997	0.636256138	-0.396311141	0.03198235	0.000000	7.79E-35
MIR663A	chr20	26188847	Promoter (<=1kb)	0.239944997	0.636256138	-0.396311141	0.034840366	0.000000	1.35E-29
MIR663A	chr20	26188850	Promoter (<=1kb)	0.239944997	0.636256138	-0.396311141	0.031976381	0.000000	7.60E-35
MIR663A	chr20	26188851	Promoter (<=1kb)	0.239944997	0.636256138	-0.396311141	0.035297961	0.000000	7.17E-29
MIR663A	chr20	26188852	Promoter (<=1kb)	0.239944997	0.636256138	-0.396311141	0.032856294	0.000000	4.33E-33
MIR663A	chr20	26188853	Promoter (<=1kb)	0.239944997	0.636256138	-0.396311141	0.035364511	0.000000	9.02E-29
MIR663A	chr20	26188863	Promoter (<=1kb)	0.239944997	0.636256138	-0.396311141	0.032764934	0.000000	2.89E-33
MIR663A	chr20	26188864	Promoter (<=1kb)	0.239944997	0.636256138	-0.396311141	0.035230615	0.000000	5.64E-29
MIR663A	chr20	26188874	Promoter (<=1kb)	0.239944997	0.636256138	-0.396311141	0.032707574	0.000000	2.26E-33
MIR663A	chr20	26188875	Promoter (<=1kb)	0.239944997	0.636256138	-0.396311141	0.03460112	0.000000	5.50E-30
MIR663A	chr20	26188880	Promoter (<=1kb)	0.239944997	0.636256138	-0.396311141	0.032759244	0.000000	2.83E-33
MIR663A	chr20	26188881	Promoter (<=1kb)	0.239944997	0.636256138	-0.396311141	0.033853904	0.000000	2.96E-31
MIR663A	chr20	26188886	Promoter (<=1kb)	0.239944997	0.636256138	-0.396311141	0.031255342	0.000000	2.11E-36
MIR663A	chr20	26188887	Promoter (<=1kb)	0.239944997	0.636256138	-0.396311141	0.031366749	0.000000	3.73E-36
MIR663A	chr20	26188888	Promoter (<=1kb)	0.239944997	0.636256138	-0.396311141	0.031090859	0.000000	9.03E-37

MIR663A	chr20	26188889	Promoter (<=1kb)	0.239944997	0.636256138	-0.396311141	0.03036195	0.000000	1.73E-38
MIR663A	chr20	26188891	Promoter (<=1kb)	0.239944997	0.636256138	-0.396311141	0.032142916	0.000000	1.67E-34
MIR663A	chr20	26188892	Promoter (<=1kb)	0.239944997	0.636256138	-0.396311141	0.034261936	0.000000	1.50E-30
MIR663A	chr20	26188896	Promoter (<=1kb)	0.239944997	0.636256138	-0.396311141	0.030646242	0.000000	8.32E-38
MIR663A	chr20	26188897	Promoter (<=1kb)	0.239944997	0.636256138	-0.396311141	0.03532477	0.000000	7.85E-29
PTGER4P2-CDK2AP2P2	chr9	66457917	Intron (uc004aec.3/uc004aec.3, intron 1 of 4)	0.376723438	0.772025244	-0.395301807	0.069063726	0.000000	1.91E-08
PTGER4P2-CDK2AP2P2	chr9	66457919	Intron (uc004aec.3/uc004aec.3, intron 1 of 4)	0.376723438	0.772025244	-0.395301807	0.068067705	0.000000	1.17E-08
PTGER4P2-CDK2AP2P2	chr9	66457931	Intron (uc004aec.3/uc004aec.3, intron 1 of 4)	0.376723438	0.772025244	-0.395301807	0.068932567	0.000000	1.80E-08
PTGER4P2-CDK2AP2P2	chr9	66457937	Intron (uc004aec.3/uc004aec.3, intron 1 of 4)	0.376723438	0.772025244	-0.395301807	0.068185266	0.000000	1.24E-08
MIR3648-1	chr21	9825955	Promoter (<=1kb)	0.298740171	0.693983752	-0.39524358	0.028770871	0.000000	1.75E-42
MIR663B	chr2	133011932	Promoter (2-3kb)	0.376800351	0.771953914	-0.395153563	0.056707304	0.000000	6.19E-12
MIR663B	chr2	133011960	Promoter (2-3kb)	0.376800351	0.771953914	-0.395153563	0.054264369	0.000000	6.47E-13
MIR663B	chr2	133011990	Promoter (2-3kb)	0.376800351	0.771953914	-0.395153563	0.055247994	0.000000	1.66E-12
MIR663B	chr2	133012005	Promoter (2-3kb)	0.376800351	0.771953914	-0.395153563	0.053412766	0.000000	2.74E-13
MIR663B	chr2	133012010	Promoter (2-3kb)	0.376800351	0.771953914	-0.395153563	0.055486848	0.000000	2.07E-12
MIR3648-1	chr21	9825954	Promoter (<=1kb)	0.298197052	0.69284041	-0.394643358	0.027091321	0.000000	1.40E-47
NA	chr21	9826699	Promoter (<=1kb)	0.275588955	0.668312325	-0.39272337	0.022007173	0.000000	1.70E-70
NA	chr21	9826700	Promoter (<=1kb)	0.275588955	0.668312325	-0.39272337	0.023494338	0.000000	4.22E-62
NA	chr21	9826704	Promoter (<=1kb)	0.275588955	0.668312325	-0.39272337	0.022159892	0.000000	1.46E-69
NA	chr21	9826705	Promoter (<=1kb)	0.275588955	0.668312325	-0.39272337	0.023287614	0.000000	3.64E-63
NA	chr21	9826708	Promoter (<=1kb)	0.275588955	0.668312325	-0.39272337	0.019592226	0.000000	2.02E-88
NA	chr21	9826709	Promoter (<=1kb)	0.275588955	0.668312325	-0.39272337	0.020860136	0.000000	2.99E-78
NA	chr21	9826716	Promoter (<=1kb)	0.275588955	0.668312325	-0.39272337	0.023445329	0.000000	2.38E-62
NA	chr21	9826717	Promoter (<=1kb)	0.275588955	0.668312325	-0.39272337	0.023665181	0.000000	2.99E-61
NA	chr21	9826718	Promoter (<=1kb)	0.275588955	0.668312325	-0.39272337	0.02130389	0.000000	4.22E-75
NA	chr21	9826719	Promoter (<=1kb)	0.275588955	0.668312325	-0.39272337	0.023677196	0.000000	3.42E-61
NA	chr21	9826722	Promoter (<=1kb)	0.275588955	0.668312325	-0.39272337	0.023368786	0.000000	9.57E-63
NA	chr21	9826723	Promoter (<=1kb)	0.275588955	0.668312325	-0.39272337	0.022818549	0.000000	1.04E-65
NA	chr21	9826650	Promoter (<=1kb)	0.280812865	0.673447591	-0.392634726	0.014423381	0.000000	6.57E-162
NA	chr21	9826654	Promoter (<=1kb)	0.281477505	0.673655253	-0.392177748	0.013662973	0.000000	7.22E-180
NA	chr21	9826651	Promoter (<=1kb)	0.281267772	0.673306345	-0.392038573	0.011717027	0.000000	7.54E-244
NA	chr21	9826652	Promoter (<=1kb)	0.281267772	0.673306345	-0.392038573	0.014093476	0.000000	5.38E-169

NA	chr21	9826653	Promoter (<=1kb)	0.281267772	0.673306345	-0.392038573	0.011771853	0.000000	1.20E-241
NA	chr21	9826658	Promoter (<=1kb)	0.281915986	0.67382645	-0.391910464	0.01386425	0.000000	1.76E-174
NA	chr21	9826655	Promoter (<=1kb)	0.282291762	0.673776328	-0.391484566	0.011485104	0.000000	5.57E-253
NA	chr21	9826656	Promoter (<=1kb)	0.282291762	0.673776328	-0.391484566	0.014240011	0.000000	4.26E-165
NA	chr21	9826657	Promoter (<=1kb)	0.282291762	0.673776328	-0.391484566	0.011746547	0.000000	5.54E-242
NA	chr21	9826649	Promoter (<=1kb)	0.280958367	0.672328308	-0.391369941	0.011365311	0.000000	4.33E-258
NA	chr21	9826637	Promoter (<=1kb)	0.288300316	0.679540174	-0.391239858	0.014937879	0.000000	5.77E-150
NA	chr21	9826661	Promoter (<=1kb)	0.281227238	0.672431946	-0.391204708	0.01156236	0.000000	2.75E-249
NA	chr21	9826648	Promoter (<=1kb)	0.282161041	0.673328237	-0.391167196	0.01338218	0.000000	1.77E-186
NA	chr21	9826644	Promoter (<=1kb)	0.284562694	0.675710396	-0.391147701	0.012611473	0.000000	8.48E-210
NA	chr21	9826645	Promoter (<=1kb)	0.284562694	0.675710396	-0.391147701	0.011279676	0.000000	1.20E-261
MIR3648-1	chr21	9826008	Promoter (<=1kb)	0.305775767	0.696616509	-0.390840742	0.015720177	0.000000	2.94E-135
NA	chr21	9826635	Promoter (<=1kb)	0.290011251	0.680850089	-0.390838839	0.023753198	0.000000	2.93E-60
NA	chr21	9826659	Promoter (<=1kb)	0.281862444	0.672660778	-0.390798335	0.011680391	0.000000	7.54E-244
NA	chr21	9826660	Promoter (<=1kb)	0.281862444	0.672660778	-0.390798335	0.011338515	0.000000	1.61E-258
NA	chr21	9826642	Promoter (<=1kb)	0.287055962	0.677841041	-0.390785078	0.013389703	0.000000	6.41E-186
NA	chr21	9826638	Promoter (<=1kb)	0.287936765	0.678650273	-0.390713508	0.012321221	0.000000	3.11E-219
NA	chr21	9826641	Promoter (<=1kb)	0.287936765	0.678650273	-0.390713508	0.015548221	0.000000	3.77E-138
NA	chr21	9826631	Promoter (<=1kb)	0.291853804	0.682560922	-0.390707118	0.024968353	0.000000	1.15E-54
NA	chr21	9826636	Promoter (<=1kb)	0.289952822	0.680377419	-0.390424597	0.012959868	0.000000	5.29E-198
MIR3648-1	chr21	9826009	Promoter (<=1kb)	0.305077709	0.694877432	-0.389799723	0.015134076	0.000000	4.49E-145
MIR3648-1	chr21	9826010	Promoter (<=1kb)	0.305077709	0.694877432	-0.389799723	0.01666202	0.000000	6.69E-120
MIR3648-1	chr21	9826011	Promoter (<=1kb)	0.305077709	0.694877432	-0.389799723	0.018814749	0.000000	2.52E-94
NA	chr21	9826379	Promoter (<=1kb)	0.297027215	0.68659599	-0.389568775	0.010723934	0.000000	7.64E-287
NA	chr21	9826380	Promoter (<=1kb)	0.297027215	0.68659599	-0.389568775	0.010916765	0.000000	7.21E-277
NA	chr21	9826378	Promoter (<=1kb)	0.297375803	0.686920415	-0.389544612	0.011115407	0.000000	3.46E-267
NA	chr21	9826920	Promoter (<=1kb)	0.272535893	0.662078884	-0.389542991	0.018060417	0.000000	4.37E-102
NA	chr21	9826921	Promoter (<=1kb)	0.272535893	0.662078884	-0.389542991	0.019942794	0.000000	4.40E-84
NA	chr21	9826632	Promoter (<=1kb)	0.291668908	0.68117195	-0.389503042	0.024621702	0.000000	7.86E-56
NA	chr21	9826382	Promoter (<=1kb)	0.297139603	0.686604896	-0.389465293	0.009539396	0.000000	0
NA	chr21	9826383	Promoter (<=1kb)	0.297139603	0.686604896	-0.389465293	0.009501258	0.000000	0
NA	chr21	9826726	Promoter (<=1kb)	0.278868531	0.668328528	-0.389459997	0.023697682	0.000000	4.02E-60

NA	chr21	9826662	Promoter (<=1kb)	0.280911304	0.670311853	-0.389400549	0.011406032	0.000000	9.54E-254
NA	chr21	9826377	Promoter (<=1kb)	0.297526264	0.686906002	-0.389379739	0.011030887	0.000000	5.75E-271
NA	chr21	9826374	Promoter (<=1kb)	0.299883722	0.689158953	-0.389275231	0.01108566	0.000000	3.32E-268
NA	chr21	9826386	Promoter (<=1kb)	0.297776682	0.686862551	-0.389085869	0.009813264	0.000000	0
NA	chr21	9827093	Promoter (<=1kb)	0.270973525	0.659984895	-0.38901137	0.020930251	0.000000	2.56E-76
NA	chr21	9827094	Promoter (<=1kb)	0.270973525	0.659984895	-0.38901137	0.021280317	0.000000	6.97E-74
GYG2P1	chrY	13470507	Distal Intergenic	0.55390516	0.942848952	-0.388943791	0.069900223	0.000000	2.09E-05
GYG2P1	chrY	13470534	Distal Intergenic	0.55390516	0.942848952	-0.388943791	0.097166213	0.000063	0.008913003
NA	chr21	9826387	Promoter (<=1kb)	0.298770667	0.687223888	-0.38845322	0.009843469	0.000000	0
NA	chr21	9826727	Promoter (<=1kb)	0.279923072	0.668333927	-0.388410855	0.0240194	0.000000	2.84E-58
NA	chr21	9826728	Promoter (<=1kb)	0.279923072	0.668333927	-0.388410855	0.023576697	0.000000	2.13E-60
NA	chr21	9826912	Promoter (<=1kb)	0.273804052	0.662087019	-0.388282967	0.019314981	0.000000	6.62E-89
NA	chr21	9826629	Promoter (<=1kb)	0.295228801	0.683449601	-0.388220799	0.0251858	0.000000	4.36E-53
NA	chr21	9826729	Promoter (<=1kb)	0.28103477	0.668339325	-0.387304555	0.022634143	0.000000	5.55E-65
NA	chr21	9826628	Promoter (<=1kb)	0.29644214	0.683453757	-0.387011618	0.025336934	0.000000	3.70E-52
NA	chr21	9826911	Promoter (<=1kb)	0.275138548	0.662095151	-0.386956603	0.018694585	0.000000	3.68E-94
NA	chr21	9826731	Promoter (<=1kb)	0.280982918	0.667728168	-0.38674525	0.019693678	0.000000	5.83E-85
NA	chr21	9826732	Promoter (<=1kb)	0.280982918	0.667728168	-0.38674525	0.020642709	0.000000	1.61E-77
LINC00273	chr16	33963535	Promoter (<=1kb)	0.379715567	0.766435487	-0.38671992	0.061567025	0.000000	6.29E-10
LINC00273	chr16	33963543	Promoter (<=1kb)	0.379715567	0.766435487	-0.38671992	0.059403039	0.000000	1.43E-10
LINC00273	chr16	33963549	Promoter (<=1kb)	0.379715567	0.766435487	-0.38671992	0.06065626	0.000000	3.43E-10
LINC00273	chr16	33963560	Promoter (1-2kb)	0.379715567	0.766435487	-0.38671992	0.059584625	0.000000	1.63E-10
LINC00273	chr16	33963581	Promoter (1-2kb)	0.379715567	0.766435487	-0.38671992	0.054027797	0.000000	1.60E-12
LINC00273	chr16	33963678	Promoter (1-2kb)	0.379715567	0.766435487	-0.38671992	0.020460712	0.000000	7.62E-79
NA	chr21	9826606	Promoter (<=1kb)	0.30549832	0.691897545	-0.386399225	0.02591599	0.000000	8.96E-50
NA	chr21	9826607	Promoter (<=1kb)	0.30549832	0.691897545	-0.386399225	0.025893224	0.000000	7.39E-50
NA	chr21	9826611	Promoter (<=1kb)	0.304278338	0.690384555	-0.386106217	0.021633304	0.000000	1.64E-70
NA	chr21	9826612	Promoter (<=1kb)	0.304278338	0.690384555	-0.386106217	0.022745868	0.000000	5.72E-64
NA	chr21	9826739	Promoter (<=1kb)	0.279242921	0.664935008	-0.385692086	0.023948595	0.000000	8.21E-58
NA	chr21	9826614	Promoter (<=1kb)	0.30236465	0.68795253	-0.38558788	0.025211192	0.000000	2.75E-52
NA	chr21	9826615	Promoter (<=1kb)	0.30236465	0.68795253	-0.38558788	0.025448885	0.000000	2.40E-51
NA	chr21	9826620	Promoter (<=1kb)	0.30236465	0.68795253	-0.38558788	0.024382761	0.000000	8.54E-56

NA	chr21	9826621	Promoter (<=1kb)	0.30236465	0.68795253	-0.38558788	0.023387552	0.000000	1.74E-60
NA	chr21	9826603	Promoter (<=1kb)	0.308750061	0.694327528	-0.385577467	0.023643728	0.000000	3.13E-59
NA	chr21	9826625	Promoter (<=1kb)	0.299517006	0.685094469	-0.385577463	0.02326375	0.000000	4.16E-61
NA	chr21	9826626	Promoter (<=1kb)	0.299517006	0.685094469	-0.385577463	0.0236546	0.000000	3.52E-59
NA	chr21	9826392	Promoter (<=1kb)	0.301855687	0.687411955	-0.385556268	0.012307576	0.000000	5.27E-214
NA	chr21	9826393	Promoter (<=1kb)	0.301855687	0.687411955	-0.385556268	0.011954977	0.000000	1.04E-226
NA	chr21	9826590	Promoter (<=1kb)	0.310088998	0.695411733	-0.385322735	0.024525535	0.000000	4.27E-55
NA	chr21	9826591	Promoter (<=1kb)	0.310088998	0.695411733	-0.385322735	0.025509102	0.000000	4.79E-51
NA	chr21	9826595	Promoter (<=1kb)	0.310088998	0.695411733	-0.385322735	0.022451421	0.000000	2.32E-65
NA	chr21	9826596	Promoter (<=1kb)	0.310088998	0.695411733	-0.385322735	0.023082246	0.000000	6.08E-62
NA	chr21	9826597	Promoter (<=1kb)	0.310088998	0.695411733	-0.385322735	0.025569225	0.000000	8.18E-51
NA	chr21	9826598	Promoter (<=1kb)	0.310088998	0.695411733	-0.385322735	0.024467871	0.000000	2.39E-55
NA	chr21	9826602	Promoter (<=1kb)	0.310088998	0.695411733	-0.385322735	0.021572351	0.000000	1.29E-70
NA	chr21	9826398	Promoter (<=1kb)	0.303929208	0.688898344	-0.384969136	0.014034352	0.000000	2.25E-164
NA	chr21	9826740	Promoter (<=1kb)	0.279202448	0.664105489	-0.384903041	0.021889131	0.000000	1.64E-68
NA	chr21	9826396	Promoter (<=1kb)	0.303139276	0.688016515	-0.38487724	0.014619382	0.000000	1.68E-151
NA	chr21	9826397	Promoter (<=1kb)	0.303139276	0.688016515	-0.38487724	0.015602149	0.000000	3.55E-133
NA	chr21	9826908	Promoter (<=1kb)	0.278814765	0.663588232	-0.384773468	0.01915031	0.000000	7.86E-89
NA	chr21	9826909	Promoter (<=1kb)	0.277639408	0.662111412	-0.384472004	0.019606356	0.000000	1.01E-84
NA	chr21	9826742	Promoter (<=1kb)	0.278209402	0.662637907	-0.384428505	0.023355163	0.000000	2.67E-60
NA	chr21	9826743	Promoter (<=1kb)	0.277313483	0.660965617	-0.383652134	0.023000621	0.000000	7.53E-62
NA	chr21	9826744	Promoter (<=1kb)	0.277313483	0.660965617	-0.383652134	0.019460807	0.000000	1.33E-85
NA	chr21	9826745	Promoter (<=1kb)	0.277313483	0.660965617	-0.383652134	0.018491922	0.000000	1.39E-94
NA	chr21	9826755	Promoter (<=1kb)	0.277313483	0.660965617	-0.383652134	0.021931817	0.000000	8.08E-68
NA	chr21	9826756	Promoter (<=1kb)	0.277313483	0.660965617	-0.383652134	0.021018943	0.000000	1.15E-73
NA	chr21	9826757	Promoter (<=1kb)	0.277313483	0.660965617	-0.383652134	0.012222243	0.000000	7.57E-215
NA	chr21	9826265	Promoter (<=1kb)	0.322522484	0.705246948	-0.382724464	0.027231315	0.000000	2.14E-44
NA	chr21	9826266	Promoter (<=1kb)	0.322522484	0.705246948	-0.382724464	0.026566897	0.000000	1.43E-46
NA	chr21	9826272	Promoter (<=1kb)	0.322522484	0.705246948	-0.382724464	0.024024941	0.000000	1.35E-56
NA	chr21	9826273	Promoter (<=1kb)	0.322522484	0.705246948	-0.382724464	0.024648458	0.000000	7.56E-54
NA	chr21	9826904	Promoter (<=1kb)	0.283833955	0.66581663	-0.381982675	0.01949117	0.000000	1.26E-84
NA	chr21	9826905	Promoter (<=1kb)	0.282568888	0.664401478	-0.38183259	0.0200984	0.000000	1.24E-79

NA	chr21	9826853	Promoter (<=1kb)	0.284450821	0.665559458	-0.381108636	0.016785286	0.000000	5.32E-113
NA	chr21	9826171	Promoter (<=1kb)	0.319881846	0.700919549	-0.381037704	0.022473497	0.000000	7.97E-64
NA	chr21	9826854	Promoter (<=1kb)	0.28541063	0.66619672	-0.38078609	0.018703821	0.000000	3.82E-91
NA	chr21	9826855	Promoter (<=1kb)	0.28541063	0.66619672	-0.38078609	0.016766504	0.000000	4.70E-113
NA	chr21	9826856	Promoter (<=1kb)	0.28541063	0.66619672	-0.38078609	0.018265337	0.000000	1.84E-95
NA	chr21	9826170	Promoter (<=1kb)	0.320500094	0.701160626	-0.380660532	0.023223265	0.000000	8.06E-60
NA	chr21	9826259	Promoter (<=1kb)	0.327063158	0.707479755	-0.380416597	0.024838391	0.000000	1.99E-52
NA	chr21	9826251	Promoter (<=1kb)	0.328547059	0.708815464	-0.380268406	0.027331906	0.000000	1.55E-43
NA	chr21	9826252	Promoter (<=1kb)	0.328547059	0.708815464	-0.380268406	0.026610248	0.000000	7.53E-46
NA	chr21	9826255	Promoter (<=1kb)	0.328547059	0.708815464	-0.380268406	0.027729326	0.000000	2.43E-42
NA	chr21	9826256	Promoter (<=1kb)	0.328547059	0.708815464	-0.380268406	0.027453555	0.000000	3.64E-43
NA	chr21	9826258	Promoter (<=1kb)	0.328547059	0.708815464	-0.380268406	0.025840964	0.000000	1.59E-48
NA	chr21	9826179	Promoter (<=1kb)	0.320278956	0.700314828	-0.380035872	0.023397223	0.000000	8.97E-59
NA	chr21	9826180	Promoter (<=1kb)	0.320278956	0.700314828	-0.380035872	0.022871311	0.000000	2.14E-61
NA	chr21	9826181	Promoter (<=1kb)	0.320278956	0.700314828	-0.380035872	0.022897624	0.000000	2.91E-61
NA	chr21	9826182	Promoter (<=1kb)	0.320278956	0.700314828	-0.380035872	0.022417477	0.000000	8.24E-64
NA	chr21	9826900	Promoter (<=1kb)	0.288750013	0.668471819	-0.379721806	0.019196049	0.000000	3.59E-86
NA	chr21	9826901	Promoter (<=1kb)	0.287441723	0.666977891	-0.379536168	0.020814221	0.000000	1.59E-73
NA	chr21	9826896	Promoter (<=1kb)	0.290662146	0.669845191	-0.379183045	0.019068049	0.000000	4.76E-87
NA	chr21	9826897	Promoter (<=1kb)	0.290662146	0.669845191	-0.379183045	0.020670779	0.000000	2.20E-74
NA	chr21	9826186	Promoter (<=1kb)	0.320351803	0.699363182	-0.379011379	0.023373415	0.000000	1.39E-58
NA	chr21	9826216	Promoter (<=1kb)	0.328847725	0.70780858	-0.378960855	0.020632289	0.000000	1.44E-74
NA	chr21	9826974	Promoter (<=1kb)	0.274620733	0.653557749	-0.378937016	0.011588017	0.000000	4.80E-233
NA	chr21	9826975	Promoter (<=1kb)	0.274620733	0.653557749	-0.378937016	0.011040931	0.000000	2.06E-256
NA	chr21	9826976	Promoter (<=1kb)	0.274620733	0.653557749	-0.378937016	0.01145208	0.000000	1.41E-238
NA	chr21	9826156	Promoter (<=1kb)	0.320695988	0.699632617	-0.378936629	0.023127653	0.000000	8.97E-60
NA	chr21	9826157	Promoter (<=1kb)	0.320695988	0.699632617	-0.378936629	0.022408328	0.000000	1.68E-63
NA	chr21	9826159	Promoter (<=1kb)	0.320695988	0.699632617	-0.378936629	0.025280232	0.000000	2.74E-50
NA	chr21	9826160	Promoter (<=1kb)	0.320695988	0.699632617	-0.378936629	0.025045465	0.000000	3.31E-51
NA	chr21	9826859	Promoter (<=1kb)	0.286461297	0.665342583	-0.378881286	0.01835774	0.000000	1.26E-93
NA	chr21	9826860	Promoter (<=1kb)	0.286461297	0.665342583	-0.378881286	0.019063011	0.000000	5.80E-87
NA	chr21	9826863	Promoter (<=1kb)	0.288537069	0.667344039	-0.37880697	0.018241769	0.000000	9.66E-95

NA	chr21	9826864	Promoter (<=1kb)	0.288537069	0.667344039	-0.37880697	0.018548892	0.000000	1.06E-91
NA	chr21	9826187	Promoter (<=1kb)	0.321417153	0.699993793	-0.37857664	0.022693429	0.000000	7.30E-62
NA	chr21	9826189	Promoter (<=1kb)	0.321417153	0.699993793	-0.37857664	0.023372565	0.000000	1.85E-58
NA	chr21	9826893	Promoter (<=1kb)	0.292206098	0.670762045	-0.378555948	0.020953101	0.000000	3.34E-72
NA	chr21	9826215	Promoter (<=1kb)	0.328045423	0.706555271	-0.378509848	0.021542761	0.000000	2.09E-68
NA	chr21	9826212	Promoter (<=1kb)	0.326895558	0.705400002	-0.378504444	0.023333593	0.000000	1.26E-58
NA	chr21	9826211	Promoter (<=1kb)	0.325390487	0.703561128	-0.378170642	0.022786675	0.000000	2.95E-61
NA	chr21	9826190	Promoter (<=1kb)	0.320573934	0.698668586	-0.378094652	0.022387583	0.000000	2.42E-63
NA	chr21	9826194	Promoter (<=1kb)	0.320573934	0.698668586	-0.378094652	0.021196916	0.000000	1.95E-70
NA	chr21	9826195	Promoter (<=1kb)	0.320573934	0.698668586	-0.378094652	0.021071549	0.000000	3.03E-71
NA	chr21	9826198	Promoter (<=1kb)	0.320573934	0.698668586	-0.378094652	0.018730236	0.000000	1.24E-89
NA	chr21	9826199	Promoter (<=1kb)	0.320573934	0.698668586	-0.378094652	0.017846145	0.000000	1.53E-98
NA	chr21	9826201	Promoter (<=1kb)	0.320573934	0.698668586	-0.378094652	0.022841168	0.000000	5.86E-61
NA	chr21	9826202	Promoter (<=1kb)	0.320573934	0.698668586	-0.378094652	0.022559982	0.000000	2.06E-62
NA	chr21	9826203	Promoter (<=1kb)	0.320573934	0.698668586	-0.378094652	0.021249209	0.000000	4.21E-70
NA	chr21	9826204	Promoter (<=1kb)	0.320573934	0.698668586	-0.378094652	0.023127052	0.000000	1.61E-59
LINC00273	chr16	33963738	Promoter (1-2kb)	0.379651645	0.757613545	-0.377961901	0.050091605	0.000000	9.05E-14
NA	chr21	9826206	Promoter (<=1kb)	0.3222675	0.699859724	-0.377592224	0.021998118	0.000000	2.25E-65
NA	chr21	9826207	Promoter (<=1kb)	0.3222675	0.699859724	-0.377592224	0.022702886	0.000000	1.66E-61
NA	chr21	9826208	Promoter (<=1kb)	0.3222675	0.699859724	-0.377592224	0.022934148	0.000000	2.51E-60
NA	chr21	9826205	Promoter (<=1kb)	0.321894815	0.699426465	-0.37753165	0.022389369	0.000000	3.74E-63
NA	chr21	9826892	Promoter (<=1kb)	0.293416312	0.670768868	-0.377352557	0.018877851	0.000000	6.09E-88
NA	chr21	9826977	Promoter (<=1kb)	0.274943387	0.651751437	-0.37680805	0.01124541	0.000000	1.56E-244
NA	chr21	9826873	Promoter (<=1kb)	0.291858598	0.668385216	-0.376526617	0.01795371	0.000000	1.40E-96
NA	chr21	9826874	Promoter (<=1kb)	0.291858598	0.668385216	-0.376526617	0.019379266	0.000000	3.27E-83
NA	chr21	9826877	Promoter (<=1kb)	0.294014413	0.670327715	-0.376313302	0.017632559	0.000000	5.65E-100
NA	chr21	9826878	Promoter (<=1kb)	0.294014413	0.670327715	-0.376313302	0.019841411	0.000000	2.24E-79
NA	chr21	9826888	Promoter (<=1kb)	0.295891968	0.671185985	-0.375294018	0.021158613	0.000000	1.13E-69
NA	chr21	9826887	Promoter (<=1kb)	0.297033162	0.671839532	-0.37480637	0.018881649	0.000000	9.32E-87
NA	chr21	9826881	Promoter (<=1kb)	0.296037982	0.670818467	-0.374780485	0.018058521	0.000000	1.23E-94
NA	chr21	9826885	Promoter (<=1kb)	0.297746946	0.67249392	-0.374746974	0.019268027	0.000000	2.24E-83
NA	chr21	9826411	Promoter (<=1kb)	0.31835151	0.692749514	-0.374398004	0.020949351	0.000000	1.09E-70

NA	chr21	9826882	Promoter (<=1kb)	0.29614424	0.670222313	-0.374078072	0.01980413	0.000000	9.39E-79
NA	chr21	9826979	Promoter (<=1kb)	0.27573066	0.649433414	-0.373702755	0.011748078	0.000000	1.38E-220
NA	chr21	9826886	Promoter (<=1kb)	0.298299415	0.67184611	-0.373546695	0.021471839	0.000000	4.24E-67
NA	chr21	9826412	Promoter (<=1kb)	0.319307511	0.6927527	-0.373445188	0.019862981	0.000000	4.75E-78
NA	chr21	9826414	Promoter (<=1kb)	0.319307511	0.6927527	-0.373445188	0.021712315	0.000000	1.25E-65
NA	chr21	9826415	Promoter (<=1kb)	0.319307511	0.6927527	-0.373445188	0.022436384	0.000000	1.35E-61
SEC22B	chr1	145092331	Intron (uc021ott.2/100288142, intron 53 of 131)	0.299864217	0.672351444	-0.372487227	0.08343527	0.000008	0.003213217
SEC22B	chr1	145092345	Intron (uc021ott.2/100288142, intron 53 of 131)	0.299864217	0.672351444	-0.372487227	0.083639461	0.000008	0.003213217
NA	chr21	9826421	Promoter (<=1kb)	0.319579035	0.691974053	-0.372395018	0.022330175	0.000000	7.91E-62
NA	chr21	9826980	Promoter (<=1kb)	0.275167033	0.647250113	-0.37208308	0.025604527	0.000000	2.32E-47
NA	chr21	9826422	Promoter (<=1kb)	0.320358871	0.6911899	-0.370831029	0.022538705	0.000000	2.97E-60
NA	chr21	9826987	Promoter (<=1kb)	0.272367506	0.642408754	-0.370041249	0.024882364	0.000000	1.58E-49
NA	chr21	9826988	Promoter (<=1kb)	0.272367506	0.642408754	-0.370041249	0.025463068	0.000000	2.31E-47
NA	chr21	9826990	Promoter (<=1kb)	0.272691459	0.64063236	-0.367940901	0.026185376	0.000000	2.23E-44
NA	chr21	9826436	Promoter (<=1kb)	0.324750893	0.690672307	-0.365921414	0.023223046	0.000000	2.09E-55
NA	chr21	9826991	Promoter (<=1kb)	0.272906685	0.637985547	-0.365078862	0.026352073	0.000000	3.52E-43
NA	chr21	9826992	Promoter (<=1kb)	0.272906685	0.637985547	-0.365078862	0.025339555	0.000000	1.41E-46
NA	chr21	9826437	Promoter (<=1kb)	0.325655779	0.690372617	-0.364716838	0.021123665	0.000000	4.10E-66
NA	chr21	9826993	Promoter (<=1kb)	0.27396232	0.636482217	-0.362519897	0.025972549	0.000000	8.31E-44
NA	chr21	9826440	Promoter (<=1kb)	0.327127279	0.689424814	-0.362297535	0.021890739	0.000000	6.13E-61
SULF2	chr20	46523994	Distal Intergenic	0.256498909	0.614586868	-0.358087959	0.073479803	0.000001	0.000187687
SULF2	chr20	46524005	Distal Intergenic	0.256498909	0.614586868	-0.358087959	0.073253467	0.000001	0.000187687
SULF2	chr20	46524080	Distal Intergenic	0.256498909	0.614586868	-0.358087959	0.100642291	0.000374	0.031947189
NOTCH2NLA	chr1	145209564	Promoter (<=1kb)	0.023082877	0.379278951	-0.356196074	0.065607333	0.000000	1.02E-07
NOTCH2NLA	chr1	145209575	Promoter (<=1kb)	0.023082877	0.379278951	-0.356196074	0.065332781	0.000000	9.00E-08
MIR663AHG	chr20	26190077	Promoter (<=1kb)	0.269859741	0.618242621	-0.34838288	0.072068813	0.000001	2.33E-06
MIR663AHG	chr20	26190088	Promoter (<=1kb)	0.269859741	0.618242621	-0.34838288	0.071909845	0.000001	2.21E-06
MIR663AHG	chr20	26190092	Promoter (<=1kb)	0.269859741	0.618242621	-0.34838288	0.070512869	0.000001	1.37E-06
MIR663AHG	chr20	26190098	Promoter (<=1kb)	0.269859741	0.618242621	-0.34838288	0.071395967	0.000001	1.86E-06
MIR663AHG	chr20	26190218	Promoter (<=1kb)	0.269859741	0.618242621	-0.34838288	0.036017493	0.000000	8.50E-22
MIR663AHG	chr20	26190219	Promoter (<=1kb)	0.269859741	0.618242621	-0.34838288	0.032892992	0.000000	7.38E-26
MIR663AHG	chr20	26190223	Promoter (<=1kb)	0.269859741	0.618242621	-0.34838288	0.039122536	0.000000	1.14E-18

MIR663AHG	chr20	26190224	Promoter (<=1kb)	0.269859741	0.618242621	-0.34838288	0.030728578	0.000000	2.08E-29
MIR663AHG	chr20	26190234	Promoter (<=1kb)	0.269859741	0.618242621	-0.34838288	0.037167967	0.000000	1.50E-20
MIR663AHG	chr20	26190235	Promoter (<=1kb)	0.269859741	0.618242621	-0.34838288	0.030289869	0.000000	3.18E-30
MIR663AHG	chr20	26190238	Promoter (<=1kb)	0.269859741	0.618242621	-0.34838288	0.041748008	0.000000	1.48E-16
MIR663AHG	chr20	26190239	Promoter (<=1kb)	0.269859741	0.618242621	-0.34838288	0.030804109	0.000000	2.84E-29
MIR663AHG	chr20	26190245	Promoter (<=1kb)	0.269859741	0.618242621	-0.34838288	0.02912631	0.000000	1.45E-32
MIR3648-1	chr21	9825862	Promoter (<=1kb)	0.353233225	0.698222626	-0.344989401	0.034394077	0.000000	2.45E-23
MIR3648-1	chr21	9825863	Promoter (<=1kb)	0.353233225	0.698222626	-0.344989401	0.03173362	0.000000	3.69E-27
MIR3648-1	chr21	9825864	Promoter (<=1kb)	0.353233225	0.698222626	-0.344989401	0.028727225	0.000000	8.16E-33
MIR3648-1	chr21	9825865	Promoter (<=1kb)	0.353233225	0.698222626	-0.344989401	0.029654444	0.000000	6.90E-31
MIR3648-1	chr21	9825871	Promoter (<=1kb)	0.353233225	0.698222626	-0.344989401	0.026473105	0.000000	2.27E-38
MIR3648-1	chr21	9825872	Promoter (<=1kb)	0.353233225	0.698222626	-0.344989401	0.032404094	0.000000	4.14E-26
MIR3648-1	chr21	9825834	Promoter (<=1kb)	0.360738588	0.698566352	-0.337827764	0.020332495	0.000000	2.17E-61
MIR3648-1	chr21	9825835	Promoter (<=1kb)	0.360738588	0.698566352	-0.337827764	0.020585271	0.000000	5.85E-60
MIR3648-1	chr21	9825840	Promoter (<=1kb)	0.360738588	0.698566352	-0.337827764	0.031408955	0.000000	1.29E-26
MIR3648-1	chr21	9825841	Promoter (<=1kb)	0.360738588	0.698566352	-0.337827764	0.035810231	0.000000	8.48E-21
MIR3648-1	chr21	9825843	Promoter (<=1kb)	0.360738588	0.698566352	-0.337827764	0.034121753	0.000000	9.02E-23
MIR3648-1	chr21	9825844	Promoter (<=1kb)	0.360738588	0.698566352	-0.337827764	0.030828897	0.000000	1.43E-27
MIR3648-1	chr21	9825846	Promoter (<=1kb)	0.360738588	0.698566352	-0.337827764	0.033627144	0.000000	2.10E-23
MIR3648-1	chr21	9825847	Promoter (<=1kb)	0.360738588	0.698566352	-0.337827764	0.031556301	0.000000	2.21E-26
MIR3648-1	chr21	9825833	Promoter (<=1kb)	0.365676525	0.70111741	-0.335440885	0.020854069	0.000000	1.13E-57
MIR663B	chr2	133012697	Promoter (1-2kb)	0.356435379	0.691212598	-0.334777219	0.039919088	0.000000	1.04E-16
MIR663B	chr2	133012707	Promoter (1-2kb)	0.356435379	0.691212598	-0.334777219	0.044676431	0.000000	1.34E-13
MIR663B	chr2	133012718	Promoter (1-2kb)	0.356435379	0.691212598	-0.334777219	0.042175762	0.000000	4.23E-15
MIR663B	chr2	133012729	Promoter (1-2kb)	0.356435379	0.691212598	-0.334777219	0.046947164	0.000000	1.94E-12
MIR663B	chr2	133012735	Promoter (1-2kb)	0.356435379	0.691212598	-0.334777219	0.044608908	0.000000	1.23E-13
MIR663B	chr2	133012743	Promoter (1-2kb)	0.356435379	0.691212598	-0.334777219	0.046153365	0.000000	7.96E-13
MIR663B	chr2	133012750	Promoter (1-2kb)	0.356435379	0.691212598	-0.334777219	0.0458201	0.000000	5.41E-13
MIR663B	chr2	133012788	Promoter (1-2kb)	0.356435379	0.691212598	-0.334777219	0.043584339	0.000000	3.17E-14
MIR663B	chr2	133012829	Promoter (1-2kb)	0.356435379	0.691212598	-0.334777219	0.04946537	0.000000	2.50E-11
TSPO2	chr6	41010317	Promoter (<=1kb)	0.419180003	0.751511733	-0.33233173	0.127689814	0.009251	0.014836348
SEC22B	chr1	145092304	Intron (uc021ott.2/100288142, intron 53 of 131)	0.498519154	0.830563584	-0.33204443	0.050953697	0.000000	2.05E-08

SEC22B	chr1	145092330	Intron (uc021ott.2/100288142, intron 53 of 131)	0.498519154	0.830563584	-0.33204443	0.048978047	0.000000	5.95E-09
SEC22B	chr1	145092344	Intron (uc021ott.2/100288142, intron 53 of 131)	0.498519154	0.830563584	-0.33204443	0.049128168	0.000000	5.95E-09
MIR3648-1	chr21	9825832	Promoter (<=1kb)	0.369970213	0.70162064	-0.331650427	0.018823031	0.000000	8.88E-69
PMF1	chr1	156186530	Intron (uc009wru.2/11243, intron 1 of 6)	0.371639652	0.692398134	-0.320758483	0.028703532	0.000000	1.28E-28
PMF1	chr1	156186531	Intron (uc009wru.2/11243, intron 1 of 6)	0.371639652	0.692398134	-0.320758483	0.030436588	0.000000	1.29E-25
PMF1	chr1	156186535	Intron (uc009wru.2/11243, intron 1 of 6)	0.371639652	0.692398134	-0.320758483	0.029939068	0.000000	2.03E-26
PMF1	chr1	156186506	Intron (uc009wru.2/11243, intron 1 of 6)	0.369353113	0.689326584	-0.319973471	0.02642021	0.000000	2.42E-33
PMF1	chr1	156186507	Intron (uc009wru.2/11243, intron 1 of 6)	0.369353113	0.689326584	-0.319973471	0.023346926	0.000000	2.72E-42
PMF1	chr1	156186508	Intron (uc009wru.2/11243, intron 1 of 6)	0.369353113	0.689326584	-0.319973471	0.02927816	0.000000	1.97E-27
PMF1	chr1	156186536	Intron (uc009wru.2/11243, intron 1 of 6)	0.374486366	0.694071587	-0.319585221	0.030407744	0.000000	1.74E-25
PMF1	chr1	156186539	Intron (uc009wru.2/11243, intron 1 of 6)	0.374486366	0.694071587	-0.319585221	0.027256228	0.000000	2.38E-31
PMF1	chr1	156186359	Intron (uc009wru.2/11243, intron 1 of 6)	0.361205101	0.68027549	-0.31907039	0.024457235	0.000000	1.89E-38
PMF1	chr1	156186375	Intron (uc009wru.2/11243, intron 1 of 6)	0.361205101	0.68027549	-0.31907039	0.027018301	0.000000	8.84E-32
PMF1	chr1	156186376	Intron (uc009wru.2/11243, intron 1 of 6)	0.361205101	0.68027549	-0.31907039	0.027655289	0.000000	2.11E-30
PMF1	chr1	156186377	Intron (uc009wru.2/11243, intron 1 of 6)	0.361205101	0.68027549	-0.31907039	0.030124838	0.000000	7.38E-26
PMF1	chr1	156186378	Intron (uc009wru.2/11243, intron 1 of 6)	0.361205101	0.68027549	-0.31907039	0.031591697	0.000000	1.22E-23
PMF1	chr1	156186379	Intron (uc009wru.2/11243, intron 1 of 6)	0.361205101	0.68027549	-0.31907039	0.025797559	0.000000	1.04E-34
PMF1	chr1	156186380	Intron (uc009wru.2/11243, intron 1 of 6)	0.361205101	0.68027549	-0.31907039	0.026192158	0.000000	1.02E-33
PMF1	chr1	156186383	Intron (uc009wru.2/11243, intron 1 of 6)	0.361205101	0.68027549	-0.31907039	0.024604522	0.000000	5.21E-38
PMF1	chr1	156186384	Intron (uc009wru.2/11243, intron 1 of 6)	0.361205101	0.68027549	-0.31907039	0.023709208	0.000000	7.96E-41
PMF1	chr1	156186387	Intron (uc009wru.2/11243, intron 1 of 6)	0.361205101	0.68027549	-0.31907039	0.025129929	0.000000	1.70E-36
PMF1	chr1	156186388	Intron (uc009wru.2/11243, intron 1 of 6)	0.361205101	0.68027549	-0.31907039	0.025034302	0.000000	9.18E-37
PMF1	chr1	156186442	Intron (uc009wru.2/11243, intron 1 of 6)	0.361205101	0.68027549	-0.31907039	0.019055662	0.000000	2.64E-62
PMF1	chr1	156186451	Intron (uc009wru.2/11243, intron 1 of 6)	0.361205101	0.68027549	-0.31907039	0.028422485	0.000000	7.28E-29
PMF1	chr1	156186454	Intron (uc009wru.2/11243, intron 1 of 6)	0.361205101	0.68027549	-0.31907039	0.027785439	0.000000	3.92E-30
PMF1	chr1	156186472	Intron (uc009wru.2/11243, intron 1 of 6)	0.361205101	0.68027549	-0.31907039	0.026722286	0.000000	1.86E-32
PMF1	chr1	156186476	Intron (uc009wru.2/11243, intron 1 of 6)	0.361205101	0.68027549	-0.31907039	0.029201797	0.000000	2.02E-27
PMF1	chr1	156186483	Intron (uc009wru.2/11243, intron 1 of 6)	0.361205101	0.68027549	-0.31907039	0.029627258	0.000000	1.12E-26
PMF1	chr1	156186540	Intron (uc009wru.2/11243, intron 1 of 6)	0.377405194	0.696191769	-0.318786574	0.026819023	0.000000	3.53E-32
PMF1	chr1	156186548	Intron (uc009wru.2/11243, intron 1 of 6)	0.377405194	0.696191769	-0.318786574	0.030993753	0.000000	1.82E-24
PMF1	chr1	156186549	Intron (uc009wru.2/11243, intron 1 of 6)	0.377405194	0.696191769	-0.318786574	0.030052994	0.000000	6.25E-26
PMF1	chr1	156186554	Intron (uc009wru.2/11243, intron 1 of 6)	0.377405194	0.696191769	-0.318786574	0.030011435	0.000000	5.36E-26

PMF1	chr1	156186555	Intron (uc009wru.2/11243, intron 1 of 6)	0.377405194	0.696191769	-0.318786574	0.031756032	0.000000	2.26E-23
PMF1	chr1	156186560	Intron (uc009wru.2/11243, intron 1 of 6)	0.377405194	0.696191769	-0.318786574	0.029853466	0.000000	2.96E-26
PMF1	chr1	156186561	Intron (uc009wru.2/11243, intron 1 of 6)	0.377405194	0.696191769	-0.318786574	0.029011033	0.000000	1.03E-27
PMF1	chr1	156186572	Intron (uc009wru.2/11243, intron 1 of 6)	0.377405194	0.696191769	-0.318786574	0.029888603	0.000000	3.37E-26
PMF1	chr1	156186573	Intron (uc009wru.2/11243, intron 1 of 6)	0.377405194	0.696191769	-0.318786574	0.031471936	0.000000	9.07E-24
PMF1	chr1	156186575	Intron (uc009wru.2/11243, intron 1 of 6)	0.377405194	0.696191769	-0.318786574	0.027385565	0.000000	6.37E-31
PMF1	chr1	156186576	Intron (uc009wru.2/11243, intron 1 of 6)	0.377405194	0.696191769	-0.318786574	0.027293593	0.000000	4.03E-31
MIR3648-1	chr21	9825802	Promoter (<=1kb)	0.384140859	0.698572707	-0.314431848	0.020634664	0.000000	6.42E-52
MIR3648-1	chr21	9825793	Promoter (<=1kb)	0.392058863	0.699747962	-0.307689099	0.016508286	0.000000	9.72E-77
MIR3648-1	chr21	9825796	Promoter (<=1kb)	0.392058863	0.699747962	-0.307689099	0.016010205	0.000000	1.91E-81
MIR3648-1	chr21	9825798	Promoter (<=1kb)	0.392058863	0.699747962	-0.307689099	0.015307853	0.000000	6.80E-89
FRG1JP	chr9	68412598	Exon (uc004aew.1/uc004aew.1, exon 2 of 2)	0.402493421	0.71011243	-0.30761901	0.050477087	0.000000	2.05E-09
DLG2	chr11	85195058	Intron (uc001pak.2/1740, intron 3 of 27)	0.257495573	0.560860762	-0.303365189	0.066039932	0.000004	7.38E-06
DLG2	chr11	85195059	Intron (uc001pak.2/1740, intron 3 of 27)	0.257495573	0.560860762	-0.303365189	0.064899827	0.000003	5.03E-06
IRS4	chrX	108297787	Distal Intergenic	0.339788872	0.642368067	-0.302579195	0.060919206	0.000001	1.20E-06
IRS4	chrX	108297788	Distal Intergenic	0.339788872	0.642368067	-0.302579195	0.065204377	0.000003	5.90E-06
IRS4	chrX	108297805	Distal Intergenic	0.339788872	0.642368067	-0.302579195	0.058892375	0.000000	4.93E-07
IRS4	chrX	108297806	Distal Intergenic	0.339788872	0.642368067	-0.302579195	0.070588667	0.000018	3.06E-05
IRS4	chrX	108297814	Distal Intergenic	0.339788872	0.642368067	-0.302579195	0.061707248	0.000001	1.65E-06
IRS4	chrX	108297815	Distal Intergenic	0.339788872	0.642368067	-0.302579195	0.065179972	0.000003	5.86E-06
IRS4	chrX	108297822	Distal Intergenic	0.339788872	0.642368067	-0.302579195	0.024273615	0.000000	3.12E-35
PMF1	chr1	156186289	Intron (uc009wru.2/11243, intron 1 of 6)	0.361777812	0.663342231	-0.30156442	0.022813734	0.000000	1.96E-39
CARTPT	chr5	71146824	Distal Intergenic	0.282615315	0.584159349	-0.301544034	0.055532942	0.000000	1.02E-07
CARTPT	chr5	71146840	Distal Intergenic	0.282615315	0.584159349	-0.301544034	0.061279178	0.000001	1.52E-06
CARTPT	chr5	71146845	Distal Intergenic	0.282615315	0.584159349	-0.301544034	0.06201015	0.000001	2.02E-06
CARTPT	chr5	71146858	Distal Intergenic	0.282615315	0.584159349	-0.301544034	0.063040727	0.000002	2.97E-06
CARTPT	chr5	71146872	Distal Intergenic	0.282615315	0.584159349	-0.301544034	0.052939878	0.000000	2.24E-08
PMF1	chr1	156186285	Intron (uc009wru.2/11243, intron 1 of 6)	0.365836322	0.661697899	-0.295861577	0.023609364	0.000000	1.37E-35
LINC00960	chr3	75720199	Promoter (1-2kb)	0.327171365	0.614131029	-0.286959664	0.081895575	0.000458	0.007751351
PMF1	chr1	156186259	Intron (uc009wru.2/11243, intron 1 of 6)	0.372594915	0.659438349	-0.286843434	0.039052251	0.000000	4.07E-13
PMF1	chr1	156186260	Intron (uc009wru.2/11243, intron 1 of 6)	0.372594915	0.659438349	-0.286843434	0.040763685	0.000000	3.81E-12
ANKRD20A12P	chr1	142618854	Exon (uc001eiw.1/uc001eiw.1, exon 1 of 8)	0.603067003	0.883622155	-0.280555153	0.058725706	0.000002	3.05E-06

ANKRD20A12P	chr1	142618859	Exon (uc001eiw.1/uc001eiw.1, exon 1 of 8)	0.603067003	0.883622155	-0.280555153	0.058763639	0.000002	3.09E-06
ANKRD20A12P	chr1	142618864	Exon (uc001eiw.1/uc001eiw.1, exon 1 of 8)	0.603067003	0.883622155	-0.280555153	0.058725706	0.000002	3.05E-06
ANKRD20A12P	chr1	142618870	Exon (uc001eiw.1/uc001eiw.1, exon 1 of 8)	0.603067003	0.883622155	-0.280555153	0.058342635	0.000002	2.63E-06
ANKRD20A12P	chr1	142618882	Exon (uc001eiw.1/uc001eiw.1, exon 1 of 8)	0.603067003	0.883622155	-0.280555153	0.058082243	0.000001	2.37E-06
PMF1	chr1	156186238	Intron (uc009wru.2/11243, intron 1 of 6)	0.382814901	0.657486885	-0.274671984	0.037565745	0.000000	5.21E-13
PMF1	chr1	156186239	Intron (uc009wru.2/11243, intron 1 of 6)	0.382814901	0.657486885	-0.274671984	0.039959333	0.000000	1.20E-11
PMF1	chr1	156186251	Intron (uc009wru.2/11243, intron 1 of 6)	0.382814901	0.657486885	-0.274671984	0.033994167	0.000000	1.34E-15
PMF1	chr1	156186252	Intron (uc009wru.2/11243, intron 1 of 6)	0.382814901	0.657486885	-0.274671984	0.033999367	0.000000	1.35E-15
LSP1P4	chr2	91777017	Distal Intergenic	0.471841875	0.744122727	-0.272280852	0.073979202	0.000233	0.000388124
LSP1P4	chr2	91777018	Distal Intergenic	0.471841875	0.744122727	-0.272280852	0.074436064	0.000254	0.000423046
LSP1P4	chr2	91777027	Distal Intergenic	0.471841875	0.744122727	-0.272280852	0.072051603	0.000157	0.000263741
LSP1P4	chr2	91777028	Distal Intergenic	0.471841875	0.744122727	-0.272280852	0.070997583	0.000126	0.000210697
MIR3648-1	chr21	9825625	Promoter (<=1kb)	0.447650039	0.708869519	-0.26121948	0.022210664	0.000000	1.56E-31
MIR3648-1	chr21	9825637	Promoter (<=1kb)	0.447650039	0.708869519	-0.26121948	0.021257525	0.000000	2.78E-34
MIR3648-1	chr21	9825663	Promoter (<=1kb)	0.447650039	0.708869519	-0.26121948	0.025193568	0.000000	7.68E-25
MIR3648-1	chr21	9825664	Promoter (<=1kb)	0.447650039	0.708869519	-0.26121948	0.024850471	0.000000	1.71E-25
MIR3648-1	chr21	9825670	Promoter (<=1kb)	0.447650039	0.708869519	-0.26121948	0.032000224	0.000000	6.76E-16
MIR3648-1	chr21	9825671	Promoter (<=1kb)	0.447650039	0.708869519	-0.26121948	0.029621251	0.000000	2.46E-18
MIR3648-1	chr21	9825673	Promoter (<=1kb)	0.447650039	0.708869519	-0.26121948	0.027448128	0.000000	3.84E-21
MIR3648-1	chr21	9825674	Promoter (<=1kb)	0.447650039	0.708869519	-0.26121948	0.026485662	0.000000	1.31E-22
MIR3648-1	chr21	9825676	Promoter (<=1kb)	0.447650039	0.708869519	-0.26121948	0.026585992	0.000000	1.90E-22
MIR3648-1	chr21	9825677	Promoter (<=1kb)	0.447650039	0.708869519	-0.26121948	0.0249076	0.000000	2.20E-25
MIR3648-1	chr21	9825679	Promoter (<=1kb)	0.447650039	0.708869519	-0.26121948	0.017482316	0.000000	5.57E-50
MIR3648-1	chr21	9825680	Promoter (<=1kb)	0.447650039	0.708869519	-0.26121948	0.01853606	0.000000	1.26E-44
LINC00273	chr16	33959613	Promoter (2-3kb)	0.718425902	0.966876794	-0.248450892	0.060003354	0.000035	0.00623472
LINC00273	chr16	33959616	Promoter (2-3kb)	0.718425902	0.966876794	-0.248450892	0.062607018	0.000072	0.007184817
FRG1JP	chr9	68412598	Exon (uc004aew.1/uc004aew.1, exon 2 of 2)	0.449501488	0.692795685	-0.243294197	0.067225726	0.000296	0.005234294
FRG1JP	chr9	68412604	Exon (uc004aew.1/uc004aew.1, exon 2 of 2)	0.449501488	0.692795685	-0.243294197	0.053805159	0.000006	0.000210555
FRG1JP	chr9	68412609	Exon (uc004aew.1/uc004aew.1, exon 2 of 2)	0.449501488	0.692795685	-0.243294197	0.054859349	0.000009	0.000289944
FRG1JP	chr9	68412612	Exon (uc004aew.1/uc004aew.1, exon 2 of 2)	0.449501488	0.692795685	-0.243294197	0.05970155	0.000046	0.001085319
MIR3648-1	chr21	9825600	Promoter (<=1kb)	0.485342872	0.722859792	-0.23751692	0.038913609	0.000000	1.94E-09
MIR3648-1	chr21	9825601	Promoter (<=1kb)	0.485342872	0.722859792	-0.23751692	0.031796567	0.000000	1.60E-13

LINC00273	chr16	33959613	Promoter (2-3kb)	0.750898556	0.974147717	-0.223249161	0.064650042	0.000554	0.025550005
MIR3648-1	chr21	9825568	Promoter (<=1kb)	0.51953973	0.734627244	-0.215087514	0.04128235	0.000000	3.35E-07
MIR3648-1	chr21	9825569	Promoter (<=1kb)	0.51953973	0.734627244	-0.215087514	0.038855908	0.000000	5.62E-08
MIR3648-1	chr21	9825572	Promoter (<=1kb)	0.51953973	0.734627244	-0.215087514	0.033775142	0.000000	3.59E-10
MIR3648-1	chr21	9825573	Promoter (<=1kb)	0.51953973	0.734627244	-0.215087514	0.038620978	0.000000	4.65E-08
MIR3648-1	chr21	9825575	Promoter (<=1kb)	0.51953973	0.734627244	-0.215087514	0.04004684	0.000000	1.41E-07
MIR3648-1	chr21	9825576	Promoter (<=1kb)	0.51953973	0.734627244	-0.215087514	0.035735345	0.000000	3.27E-09
MIR3648-1	chr21	9825579	Promoter (<=1kb)	0.51953973	0.734627244	-0.215087514	0.033558397	0.000000	2.76E-10
MIR3648-1	chr21	9825580	Promoter (<=1kb)	0.51953973	0.734627244	-0.215087514	0.036530743	0.000000	7.26E-09
MIR3648-1	chr21	9825550	Promoter (<=1kb)	0.519542749	0.733160442	-0.213617693	0.040343441	0.000000	2.12E-07
MIR3648-1	chr21	9825551	Promoter (<=1kb)	0.519542749	0.733160442	-0.213617693	0.037861397	0.000000	3.06E-08
MIR3648-1	chr21	9825582	Promoter (<=1kb)	0.522190321	0.735684707	-0.213494386	0.044504939	0.000002	2.78E-06
MIR3648-1	chr21	9825583	Promoter (<=1kb)	0.524698072	0.737580074	-0.212882002	0.030929963	0.000000	1.13E-11
MIR3648-1	chr21	9825490	Promoter (<=1kb)	0.519551811	0.727714054	-0.208162243	0.032294911	0.000000	2.18E-10
MIR3648-1	chr21	9825491	Promoter (<=1kb)	0.519551811	0.727714054	-0.208162243	0.044092477	0.000002	4.01E-06
MIR3648-1	chr21	9825495	Promoter (<=1kb)	0.519551811	0.727714054	-0.208162243	0.038834632	0.000000	1.49E-07
MIR3648-1	chr21	9825496	Promoter (<=1kb)	0.519551811	0.727714054	-0.208162243	0.036414946	0.000000	1.99E-08
MIR3648-1	chr21	9825498	Promoter (<=1kb)	0.519551811	0.727714054	-0.208162243	0.035492899	0.000000	8.32E-09
MIR3648-1	chr21	9825499	Promoter (<=1kb)	0.519551811	0.727714054	-0.208162243	0.039217776	0.000000	1.98E-07
LINC00273	chr16	33959613	Promoter (2-3kb)	0.764669837	0.965936618	-0.201266781	0.05348707	0.000168	0.004118445
LINC00273	chr16	33959616	Promoter (2-3kb)	0.764669837	0.965936618	-0.201266781	0.055703374	0.000302	0.005686152
LINC00273	chr16	33959613	Promoter (2-3kb)	0.778067976	0.976999298	-0.198931322	0.05128469	0.000105	0.007387627
LINC00273	chr16	33959616	Promoter (2-3kb)	0.778067976	0.976999298	-0.198931322	0.051986196	0.000130	0.007387627
MUC2	chr11	1093344	Exon (uc001lsx.1/4583, exon 31 of 51)	0.656584363	0.853893206	-0.197308843	0.051739392	0.000137	0.010891925
MUC2	chr11	1093368	Exon (uc001lsx.1/4583, exon 31 of 51)	0.656584363	0.853893206	-0.197308843	0.051274717	0.000119	0.010516144
MIR663A	chr20	26188698	Promoter (<=1kb)	0.329545924	0.481260583	-0.151714659	0.027687613	0.000000	8.18E-06
MIR663A	chr20	26188699	Promoter (<=1kb)	0.329545924	0.481260583	-0.151714659	0.014790238	0.000000	6.19E-22
MIR663A	chr20	26188715	Promoter (<=1kb)	0.329545924	0.481260583	-0.151714659	0.024228515	0.000000	1.44E-07
MIR663A	chr20	26188718	Promoter (<=1kb)	0.329545924	0.481260583	-0.151714659	0.029086344	0.000000	2.96E-05
MIR663A	chr20	26188722	Promoter (<=1kb)	0.329545924	0.481260583	-0.151714659	0.027701307	0.000000	8.18E-06
MIR663A	chr20	26188725	Promoter (<=1kb)	0.329545924	0.481260583	-0.151714659	0.029315721	0.000000	3.22E-05
MIR663A	chr20	26188729	Promoter (<=1kb)	0.329545924	0.481260583	-0.151714659	0.025269442	0.000000	5.46E-07

MIR663A	chr20	26188741	Promoter (<=1kb)	0.329545924	0.481260583	-0.151714659	0.014584369	0.000000	2.73E-22
MIR663A	chr20	26188886	Promoter (<=1kb)	0.329545924	0.481260583	-0.151714659	0.029902803	0.000000	4.91E-05
MIR663A	chr20	26188889	Promoter (<=1kb)	0.329545924	0.481260583	-0.151714659	0.031391147	0.000001	0.000117191
MIR663A	chr20	26188891	Promoter (<=1kb)	0.329545924	0.481260583	-0.151714659	0.031241512	0.000001	0.000117191
LINC00273	chr16	33963678	Promoter (1-2kb)	0.18597515	0.311298049	-0.1253229	0.013646826	0.000000	2.36E-17
NA	chr21	9827373	Promoter (1-2kb)	0.486523589	0.347435658	0.139087931	0.01796079	0.000000	2.46E-13
NA	chr21	9827407	Promoter (1-2kb)	0.486523589	0.347435658	0.139087931	0.023638888	0.000000	2.60E-08
MIR3648-1	chr21	9825490	Promoter (<=1kb)	0.345410971	0.2007072	0.14470377	0.025748515	0.000000	2.81E-07
MIR663B	chr2	133012697	Promoter (1-2kb)	0.489377706	0.333185596	0.15619211	0.031824041	0.000001	4.75E-06
MIR663B	chr2	133012788	Promoter (1-2kb)	0.489377706	0.333185596	0.15619211	0.02992502	0.000000	1.09E-06
MIR663A	chr20	26188846	Promoter (<=1kb)	0.392801798	0.234425617	0.158376181	0.034192185	0.000004	1.64E-05
MIR663A	chr20	26188886	Promoter (<=1kb)	0.392801798	0.234425617	0.158376181	0.034994192	0.000006	2.70E-05
PMF1	chr1	156186285	Intron (uc009wru.2/11243, intron 1 of 6)	0.48866874	0.326992436	0.161676304	0.019488343	0.000000	5.49E-15
CWH43	chr4	49318855	Distal Intergenic	0.576937812	0.41288232	0.164055492	0.033875715	0.000001	0.000117191
PMF1	chr1	156186289	Intron (uc009wru.2/11243, intron 1 of 6)	0.490651909	0.325906113	0.164745795	0.01905594	0.000000	5.47E-16
PMF1	chr1	156186359	Intron (uc009wru.2/11243, intron 1 of 6)	0.495201892	0.329459773	0.165742118	0.035729709	0.000004	1.63E-05
PMF1	chr1	156186375	Intron (uc009wru.2/11243, intron 1 of 6)	0.495201892	0.329459773	0.165742118	0.03475283	0.000002	9.09E-06
PMF1	chr1	156186379	Intron (uc009wru.2/11243, intron 1 of 6)	0.495201892	0.329459773	0.165742118	0.032159084	0.000000	1.53E-06
PMF1	chr1	156186380	Intron (uc009wru.2/11243, intron 1 of 6)	0.495201892	0.329459773	0.165742118	0.036837705	0.000007	3.02E-05
PMF1	chr1	156186383	Intron (uc009wru.2/11243, intron 1 of 6)	0.495201892	0.329459773	0.165742118	0.033524347	0.000001	4.06E-06
PMF1	chr1	156186384	Intron (uc009wru.2/11243, intron 1 of 6)	0.495201892	0.329459773	0.165742118	0.033914903	0.000001	5.16E-06
PMF1	chr1	156186387	Intron (uc009wru.2/11243, intron 1 of 6)	0.495201892	0.329459773	0.165742118	0.035741981	0.000004	1.63E-05
PMF1	chr1	156186388	Intron (uc009wru.2/11243, intron 1 of 6)	0.495201892	0.329459773	0.165742118	0.032296919	0.000000	1.70E-06
PMF1	chr1	156186506	Intron (uc009wru.2/11243, intron 1 of 6)	0.524586198	0.339277775	0.185308423	0.022662597	0.000000	1.08E-14
PMF1	chr1	156186507	Intron (uc009wru.2/11243, intron 1 of 6)	0.524586198	0.339277775	0.185308423	0.022811032	0.000000	1.42E-14
PMF1	chr1	156186508	Intron (uc009wru.2/11243, intron 1 of 6)	0.524586198	0.339277775	0.185308423	0.042355418	0.000012	5.27E-05
PMF1	chr1	156186540	Intron (uc009wru.2/11243, intron 1 of 6)	0.539298778	0.34731444	0.191984338	0.03813118	0.000000	2.64E-06
PMF1	chr1	156186548	Intron (uc009wru.2/11243, intron 1 of 6)	0.539298778	0.34731444	0.191984338	0.0261973	0.000000	2.16E-12
PMF1	chr1	156186549	Intron (uc009wru.2/11243, intron 1 of 6)	0.539298778	0.34731444	0.191984338	0.044751318	0.000018	7.15E-05
PMF1	chr1	156186554	Intron (uc009wru.2/11243, intron 1 of 6)	0.539298778	0.34731444	0.191984338	0.02596135	0.000000	1.41E-12
PMF1	chr1	156186555	Intron (uc009wru.2/11243, intron 1 of 6)	0.539298778	0.34731444	0.191984338	0.044599452	0.000017	6.76E-05
PMF1	chr1	156186560	Intron (uc009wru.2/11243, intron 1 of 6)	0.539298778	0.34731444	0.191984338	0.024551231	0.000000	1.44E-13

PMF1	chr1	156186561	Intron (uc009wru.2/11243, intron 1 of 6)	0.539298778	0.34731444	0.191984338	0.043906542	0.000012	5.27E-05
PMF1	chr1	156186572	Intron (uc009wru.2/11243, intron 1 of 6)	0.539298778	0.34731444	0.191984338	0.023387383	0.000000	9.11E-15
PMF1	chr1	156186573	Intron (uc009wru.2/11243, intron 1 of 6)	0.539298778	0.34731444	0.191984338	0.043102127	0.000008	3.69E-05
PMF1	chr1	156186575	Intron (uc009wru.2/11243, intron 1 of 6)	0.539298778	0.34731444	0.191984338	0.022550502	0.000000	1.25E-15
PMF1	chr1	156186576	Intron (uc009wru.2/11243, intron 1 of 6)	0.539298778	0.34731444	0.191984338	0.023568438	0.000000	1.28E-14
LSP1P4	chr2	91777069	Distal Intergenic	0.513512055	0.319539082	0.193972973	0.045322541	0.000019	0.003213217
LSP1P4	chr2	91777072	Distal Intergenic	0.513512055	0.319539082	0.193972973	0.044327103	0.000012	0.003213217
LSP1P4	chr2	91777093	Distal Intergenic	0.513512055	0.319539082	0.193972973	0.045286447	0.000018	0.003213217
PMF1	chr1	156186536	Intron (uc009wru.2/11243, intron 1 of 6)	0.539208524	0.34495338	0.194255144	0.045313327	0.000018	7.18E-05
PMF1	chr1	156186539	Intron (uc009wru.2/11243, intron 1 of 6)	0.539208524	0.34495338	0.194255144	0.020347654	0.000000	5.46E-19
CCDC144BP	chr17	18538249	Distal Intergenic	0.226911452	0.032232629	0.194678823	0.050542696	0.000117	0.007387627
CCDC144BP	chr17	18538252	Distal Intergenic	0.226911452	0.032232629	0.194678823	0.050542696	0.000117	0.007387627
CCDC144BP	chr17	18538262	Distal Intergenic	0.226911452	0.032232629	0.194678823	0.05052072	0.000116	0.007387627
CCDC144BP	chr17	18538272	Distal Intergenic	0.226911452	0.032232629	0.194678823	0.050918638	0.000132	0.007387627
PMF1	chr1	156186530	Intron (uc009wru.2/11243, intron 1 of 6)	0.539118432	0.34264476	0.196473672	0.023104118	0.000000	1.25E-15
PMF1	chr1	156186531	Intron (uc009wru.2/11243, intron 1 of 6)	0.539118432	0.34264476	0.196473672	0.045013956	0.000013	5.35E-05
PMF1	chr1	156186535	Intron (uc009wru.2/11243, intron 1 of 6)	0.539118432	0.34264476	0.196473672	0.025569566	0.000000	2.62E-13
FRG1JP	chr9	68411305	Intron (uc004aew.1/uc004aew.1, intron 1 of 1)	0.438121269	0.213320272	0.224800997	0.063790197	0.000425	0.020439274
MIR663A	chr20	26189148	Promoter (<=1kb)	0.440173403	0.203562015	0.236611387	0.032965748	0.000000	6.04E-12
MIR663A	chr20	26189159	Promoter (<=1kb)	0.440173403	0.203562015	0.236611387	0.076073582	0.001869	0.00426006
MIR663A	chr20	26189163	Promoter (<=1kb)	0.440173403	0.203562015	0.236611387	0.076205873	0.001903	0.004297058
MIR663A	chr20	26189176	Promoter (<=1kb)	0.440173403	0.203562015	0.236611387	0.075395212	0.001699	0.003939557
MIR663A	chr20	26189178	Promoter (<=1kb)	0.440173403	0.203562015	0.236611387	0.075367638	0.001693	0.003939557
MIR663A	chr20	26189181	Promoter (<=1kb)	0.440173403	0.203562015	0.236611387	0.07585567	0.001813	0.004156179
FANK1	chr10	127585237	Promoter (<=1kb)	0.350672511	0.046554313	0.304118198	0.072158491	0.000025	0.001923483
BRF1	chr14	105767329	Promoter (<=1kb)	0.438998876	0.002133683	0.436865193	0.198741915	0.027938	0.041299925
BRF1	chr14	105767339	Promoter (<=1kb)	0.438998876	0.002133683	0.436865193	0.198741915	0.027938	0.041299925
BRF1	chr14	105767349	Promoter (<=1kb)	0.438998876	0.002133683	0.436865193	0.198741915	0.027938	0.041299925
BRF1	chr14	105767357	Promoter (<=1kb)	0.438998876	0.002133683	0.436865193	0.198741915	0.027938	0.041299925
BRF1	chr14	105767360	Promoter (<=1kb)	0.438998876	0.002133683	0.436865193	0.198741915	0.027938	0.041299925
FANK1	chr10	127585208	Promoter (<=1kb)	0.454365581	0.011312639	0.443052943	0.118855033	0.000193	0.010272992
FANK1	chr10	127585237	Promoter (<=1kb)	0.454365581	0.011312639	0.443052943	0.031460363	0.000000	4.89E-42

DUX4L3	chr10	135491039	Promoter (2-3kb)	0.756056216	0.247723074	0.508333142	0.177277495	0.004138	0.016361678
DUX4L3	chr10	135491043	Promoter (2-3kb)	0.756056216	0.247723074	0.508333142	0.177398709	0.004164	0.016361678
DUX4L3	chr10	135491045	Promoter (2-3kb)	0.756056216	0.247723074	0.508333142	0.177383385	0.004160	0.016361678
DUX4L3	chr10	135491055	Promoter (2-3kb)	0.756056216	0.247723074	0.508333142	0.177277495	0.004138	0.016361678
DUX4L3	chr10	135491067	Promoter (2-3kb)	0.756056216	0.247723074	0.508333142	0.176415611	0.003958	0.016361678
SCGB1C1	chr11	189604	Distal Intergenic	0.906284211	0.268901174	0.637383038	0.149869761	0.000021	0.000793389
SCGB1C1	chr11	189606	Distal Intergenic	0.906284211	0.268901174	0.637383038	0.149647364	0.000021	0.000793389
SCGB1C1	chr11	189617	Distal Intergenic	0.906284211	0.268901174	0.637383038	0.149647364	0.000021	0.000793389
SCGB1C1	chr11	189626	Distal Intergenic	0.906284211	0.268901174	0.637383038	0.148071972	0.000017	0.000793389
SCGB1C1	chr11	189634	Distal Intergenic	0.906284211	0.268901174	0.637383038	0.148769726	0.000018	0.000793389
SCGB1C1	chr11	189655	Distal Intergenic	0.906284211	0.268901174	0.637383038	0.149264341	0.000020	0.000793389
SCGB1C1	chr11	189662	Distal Intergenic	0.906284211	0.268901174	0.637383038	0.149267463	0.000020	0.000793389
SCGB1C1	chr11	189663	Distal Intergenic	0.906284211	0.268901174	0.637383038	0.149682874	0.000021	0.000793389
FLJ36000	chr17	21904545	Promoter (<=1kb)	0.910320664	0.247723074	0.662597591	0.106338872	0.000000	8.15E-09
FLJ36000	chr17	21904546	Promoter (<=1kb)	0.910320664	0.247723074	0.662597591	0.106131409	0.000000	8.15E-09
FLJ36000	chr17	21904564	Promoter (<=1kb)	0.910320664	0.247723074	0.662597591	0.106124966	0.000000	8.15E-09
FLJ36000	chr17	21904565	Promoter (<=1kb)	0.910320664	0.247723074	0.662597591	0.106130788	0.000000	8.15E-09
FLJ36000	chr17	21904579	Promoter (<=1kb)	0.910320664	0.247723074	0.662597591	0.106362017	0.000000	8.15E-09
FLJ36000	chr17	21904580	Promoter (<=1kb)	0.910320664	0.247723074	0.662597591	0.106130788	0.000000	8.15E-09
FLJ36000	chr17	21904585	Promoter (<=1kb)	0.910320664	0.247723074	0.662597591	0.106338872	0.000000	8.15E-09
FLJ36000	chr17	21904586	Promoter (<=1kb)	0.910320664	0.247723074	0.662597591	0.106130788	0.000000	8.15E-09
FLJ36000	chr17	21904587	Promoter (<=1kb)	0.910320664	0.247723074	0.662597591	0.106338872	0.000000	8.15E-09
FLJ36000	chr17	21904588	Promoter (<=1kb)	0.910320664	0.247723074	0.662597591	0.106031799	0.000000	8.15E-09
FLJ36000	chr17	21904590	Promoter (<=1kb)	0.910320664	0.247723074	0.662597591	0.106362017	0.000000	8.15E-09
FLJ36000	chr17	21904591	Promoter (<=1kb)	0.910320664	0.247723074	0.662597591	0.106072038	0.000000	8.15E-09
FLJ36000	chr17	21904618	Promoter (<=1kb)	0.910320664	0.247723074	0.662597591	0.106362017	0.000000	8.15E-09
FLJ36000	chr17	21904619	Promoter (<=1kb)	0.910320664	0.247723074	0.662597591	0.10613191	0.000000	8.15E-09
FLJ36000	chr17	21904624	Promoter (<=1kb)	0.910320664	0.247723074	0.662597591	0.106362017	0.000000	8.15E-09
FLJ36000	chr17	21904625	Promoter (<=1kb)	0.910320664	0.247723074	0.662597591	0.10611287	0.000000	8.15E-09
SEMA6D	chr15	47660813	Intron (uc001zvw.3/80031, intron 1 of 19)	0.921218168	0.247723074	0.673495094	0.188883206	0.000363	0.002025141
SEMA6D	chr15	47660817	Intron (uc001zvw.3/80031, intron 1 of 19)	0.921218168	0.247723074	0.673495094	0.188861257	0.000362	0.002025141
SEMA6D	chr15	47660825	Intron (uc001zvw.3/80031, intron 1 of 19)	0.921218168	0.247723074	0.673495094	0.188883206	0.000363	0.002025141

SEMA6D	chr15	47660829	Intron (uc001zvw.3/80031, intron 1 of 19)	0.921218168	0.247723074	0.673495094	0.188791012	0.000361	0.002025141
RBFADN	chr18	77831331	Intron (uc010dri.2/79863, intron 2 of 2)	0.975256704	0.268901174	0.70635553	0.19287221	0.000250	0.004907567
RBFADN	chr18	77831332	Intron (uc010dri.2/79863, intron 2 of 2)	0.975256704	0.268901174	0.70635553	0.192504663	0.000243	0.004907567
RBFADN	chr18	77831351	Intron (uc010dri.2/79863, intron 2 of 2)	0.975256704	0.268901174	0.70635553	0.192999965	0.000252	0.004907567
RBFADN	chr18	77831352	Intron (uc010dri.2/79863, intron 2 of 2)	0.975256704	0.268901174	0.70635553	0.192504663	0.000243	0.004907567
DUSP22	chr6	314137	Intron (uc011dhn.1/56940, intron 3 of 6)	0.995732389	0.268901174	0.726831215	0.221853913	0.001052	0.015617615
DUSP22	chr6	314150	Intron (uc011dhn.1/56940, intron 3 of 6)	0.995732389	0.268901174	0.726831215	0.221853913	0.001052	0.015617615
